# Seasonal Shifts in Soil Microbiome Structure Are Associated with the Cultivation of the Local Runner Bean Variety around the Lake Mikri Prespa

**DOI:** 10.3390/biology11111595

**Published:** 2022-10-31

**Authors:** Evangelia Stavridou, Ioanna Karamichali, Georgios Lagiotis, Elena Patsea, Maslin Osathanunkul, Panagiotis Madesis

**Affiliations:** 1 Institute of Applied Biosciences, Centre for Research and Technology, 57001 Thessaloniki, Greece; 2 A.S. “PELEKANOS”, Epar.Od. Lemou-Vronterou, 53077 Prespes, Greece; 3 Department of Biology, Faculty of Science, Chiang Mai University, Chiang Mai 50200, Thailand; 4 Research Centre on Bioresources for Agriculture, Industry and Medicine, Chiang Mai University, Chiang Mai 50200, Thailand; 5 Laboratory of Molecular Biology of Plants, School of Agricultural Sciences, University of Thessaly, 38446 Volos, Greece

**Keywords:** soil microbiome, functional microbial diversity, leguminous crop, runner bean, soil metataxonomics

## Abstract

**Simple Summary:**

The “Prespa beans” are an important leguminous crop for the agricultural economy of the rural areas surrounding the lake Mikri Prespa. However, agricultural intensification and climate change have a negative impact on the available arable land, soil microbiome and, consequently, crop productivity. This study investigated the effects of seasonal changes on soil microbiome associated with runner bean cultivation. The results indicated that the presence of the runner bean and the soil processes affecting the carbon cycle differentially shaped the structure of the soil microbial community. Ultimately, this study highlights the importance of investigating soil microbiome in agriculture and is expected to contribute to future research for the development of novel preservation strategies for native soil ecosystem services.

**Abstract:**

Leguminous crops play a key role in food production and agroecosystem sustainability. However, climate change and agricultural intensification have a significant impact on the available arable land, soil microbiome functions, and ultimately, crop productivity. The “Prespa bean” (*Phaseolous coccineous* L.) is an important leguminous crop for the agricultural economy of the rural areas surrounding the lake, Mikri Prespa, which is of significant ecological importance. The seasonal effects on soil microbiome structure, diversity and functions associated with the runner bean cultivation were investigated using 16S rRNA amplicon sequencing. The results indicated that the presence of the runner bean differentially shaped the soil microbial community structure. The runner bean was implicated in the recruitment of specific bacteria, by favouring or excluding specific classes or even phyla. Soil functions involved in nutrient availability and carbon metabolism, among other pathways, were associated with microbiome–plant interactions. The temporal relative abundance shifts could be explained by the impact of soil organic matter, the fertilization regime, and the equilibrium in carbon metabolic processes. This research has shown the effect of runner bean cultivation on the soil microbiome which, in future, may potentially contribute to research into sustainable agricultural productivity and the protection of soil ecosystem services.

## 1. Introduction

Alternative farming practices, such as conservation agriculture, have been used to reduce such adverse environmental effects [1,2] by using closed nutrient cycle systems and incorporating perennial and leguminous plants, which positively affect soil microbial community size and activity [3]. Additionally, fallow fields have been shown to promote the establishment of biota, resulting in drastic changes in plant and microbiome diversity, as well as soil quality. Research has shown that the organic matter, nutrients, and microbial biomass present in soil is higher in fields left fallow for extended periods [4].

Soil is considered a slow-forming and non-renewable resource [5], yet it is essential for crop production. Soil microbiota play crucial roles in agricultural ecosystems due to their involvement in various soil processes and functions [3,6,7,8]. This is achieved via the modulation of organic matter decomposition, nutrient cycling, soil erosion control, and interactions with plants [9,10,11]. Such processes can greatly impact soil fertility, which subsequently supports crop production [12,13,14]. Despite the limited knowledge pertaining to the functions of the highly complex and diverse soil microbiome in the agroecosystems, there is evidence of associations between microbiota diversity and functional bio-processes, such as marker genes related to nitrification [9,13], being associated with soil multifunctionality [15,16]. Microbial communities rich in functional diversity could provide better resilience to environmental changes [3]. In this context, the reduction of microbial species richness may not affect soil ecosystem functions, considering that the same functions can be performed by different microorganisms, without disturbing plant productivity [17,18].

Microbial functions can be explained by focusing on microbial guilds (metabolically related taxa) or consortia (groups living symbiotically) instead of individual taxa [13,19], however, specific taxa may also play unique roles [20]. These unique keystone taxa play an ecologically important role by influencing microbiome structure and functioning, irrespective of their abundance [21,22], and thus can be used as indicators of microbiome compositional shifts [23]. Aside from the soil microbiota, plants also host intricate networks of microbial communities, which form complex symbiotic associations [24] and have a key role in plant performance and diversity [25,26,27]. Such plant microbiota interactions are not randomly assembled from the soil biota, but are rather the result of host-mediated recruitment signals that favour specific bacteria in the rhizosphere [28,29]. More specifically, there is a dynamic relationship between root exudates and symbiotic microbiota communities, which mutually influence symbiotic associations and modulation in the rhizosphere [20,30]. 

The symbiotic relationships of leguminous crops with soil nitrogen-fixing bacteria [31] and other endophytic [32,33] and non-endophytic bacteria [34,35,36,37] have been extensively studied in the literature, given their contribution to agricultural sustainability and productivity [38,39,40] by initiating nutrient cycling in nutrient-depleted soils [41]. Agronomically important examples of nitrogen-fixing symbiosis in the Fabaceae family, include the common bean (*Phaseolus vulgaris* L.) with *Rhizobium etli*, and lentils (*Lens culinaris* Medik.) with *Rhizobium leguminosarum* bv. *viciae*, as reviewed by Glodowska et al. [42]. The use of such legume-rhizobia symbioses in farming systems may not only improve nitrogen acquisition by non-leguminous crops [43,44], but may also potentially induce positive feedback mechanisms by reducing the use of fertilizers in the agroecosystem [45]. The influence of leguminous species on the diversity patterns of different rhizobia inhabiting the rhizosphere and the formed nodules has been demonstrated through high-throughput metagenomic sequencing [46,47]. However, the diversity and specificity of non-symbiotic microorganisms associated with the legume rhizosphere and the microbe–microbe interactions affected by the presence of a leguminous crop remain unclear.

The runner bean (*Phaseolus coccineus* L.) is a legume species with increased cold tolerance cultivated as an annual crop for dry seeds and immature green pods, especially in small-scale agriculture [48,49]. Beans, and especially the runner bean, are the only crops cultivated in the Mikri Prespa lake area, contributing substantially to the local economy. However, the intensive cultivation of beans has led to an increased pollution of the lake with phosphorous and pesticides, due to the intensification of the cultivation [50,51]. Bean crops used in intercropping management practices have been shown to reduce the risk of soil erosion [52], however, in the Prespa lake area the runner bean is used as a main crop and not as a cover crop. In this study, we explored the impact of the runner bean (*Phaseolous coccineous* L.) in the soil microbiome of different fields located around the Mikri (Small) Prespa lake in western Macedonia, Greece, based on the seasonal changes observed during (summer), before (spring), and after (autumn) the cultivation period. The analysis of the soil samples, across season and field, was carried out using 16S rRNA amplicon sequencing. The studied area is of significant ecological importance, especially the soils along the shoreline and the slopes of the mountains Triklario and Varnoundas, which constitute the Prespa National Park–NATURA 2000 and are included in the Wetlands of International Importance (Ramsar Convention). Given that leguminous crops play a key role in food production and agroecosystem sustainability worldwide, and especially in the rural areas surrounding the lake, Mikri Prespa, we aimed at addressing: (a) the temporal variability in soil microbial community at the scale of seasons (spring, summer and autumn), which corresponds to the periods prior, during, and after the cultivation of the local runner bean variety, (b) the prevalence of bacteria that have a potential role as key hub taxa and (c) an investigation into the functional diversity and its potential effect on the soil using metataxonomic and functional analyses.

## 2. Materials and Methods

### 2.1. Experimental Site and Soil Samples

**Figure 1:**
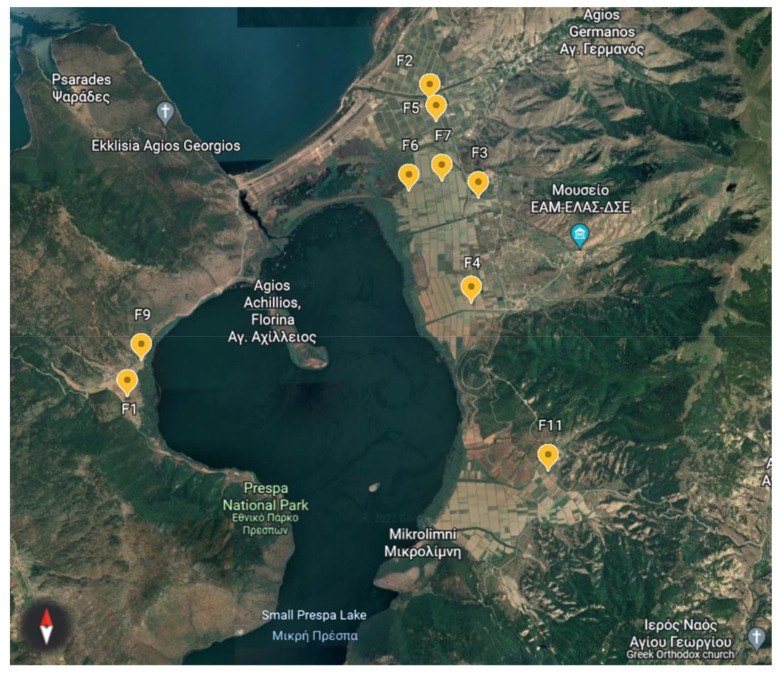
This is a figure. Schemes follow the same formatting. Google Earth, 2022. Mikri Prespa Lake: The nine different field sites (F1–F11) organised in 5 groups surrounding the Mikri Prespa Lake area indicated with orange placemarks, 1:3000.

The sampled experimental field site is located in the area around the lake, Prespa, in Florina (Northwest Greece), where Greek landraces of the runner bean are traditionally cultivated as dry beans. The legume crop was cultivated from April to November. The annual precipitation in the studied area was 730 mm. Soil samples were collected from nine different fields, in three time points (seasons/cultivation period): in March (Spring, before cultivation) with a mean precipitation and temperature of 59 mm and 5.9 °C, end of July (Summer, during cultivation) with a mean precipitation and temperature of 36 mm and 21 °C and December (Autumn, after cultivation) with a mean precipitation and temperature of 78.9 mm and 7 °C (Table S1; Figure 1) (Climatic data). The fields were left fallow (bare land) after the runner bean cultivation was resumed until the next sowing season in April, and no intercropping management practices were used. The soil texture in the area is characterised as sandy loam (75% sand, 14% silt and 11% clay), slightly acidic with 6.9 pH, a medium content in available phosphorus and a high content in available potassium. Soil sampling was performed in two locations at the centre of each field. For each location, three independent peripheral soil cores (0–60 cm depth), 1 m apart from the central point, were combined and thoroughly mixed before DNA extraction.

### 2.2. DNA Isolation, Library Preparation and Sequencing

DNA was extracted from the two independent locations in each field using the NucleoSpin Soil, Mini kit (MACHEREY-NAGEL, Düren, Germany). DNA quality and quantity were assessed with the UV-Vis Spectrophotometer Q5000 (Quawell Technology Inc., San Jose, CA, USA). The DNA samples used for sequencing were an equimolar pool of the two independent DNA extracts for each field and season (Table S1). The 16S rRNA libraries were constructed after amplification between the V3 and V4 regions of the prokaryotic ribosomal 16S RNA gene subunit using the 16S Amplicon PCR Forward [5′-TCGTCGGCAGCGTCAGATGTGTATAAGAGACAGCCTACGGGNGGCWGCAG-3′] and Reverse [5′-GTCTCGTGGGCTCGGAGATGTGTATAAGAGACAGGACTACHVGGTATCTAATCC-3′] primers (16S Metagenomics Protocol by Illumina, Part #15044223 Rev.B), containing the Illumina overhang adapter [53]. Next generation sequencing was performed in 2 × 300 bp paired-end reads with the Illumina MiSeq platform (Illumina Inc., San Diego, CA, USA).

### 2.3. Bioinformatic Analysis

The metagenomics analysis was based on the Mothur Standard Operating Procedure (SOP; https://mothur.org/wiki/miseq_sop/, accessed on 6 June 2022). The raw sequencing reads were combined per sample and analysed using the program Mothur (v.1.44.1) [54]. The sequences were organised in contigs and filtered based on sequencing quality (sequences with ambiguous bases were removed), size (Average size of contigs or 450 plus 20), and redundancy (only unique sequences were analysed). The fasta and taxonomy files from the recreated seed database (8517 bacteria, 147 archaea, and 2516 eukarya sequences, release 132) offered by Mothur and SILVA, were used for aligning the contigs to assign their taxonomy using an assignment bootstrap confidence cut-off value equal to 80%, selected based on the best practices described by the Metagenomics analysis using the Mothur Standard Operating Procedure (SOP). The aligned sequences were furthermore filtered based on their alignment quality (aligned within a selected effective for identification region set between the positions 6388 and 25,316). Unwanted lineages (Chloroplast-Mitochondria-unknown-Archaea-Eukaryota) and sequence chimeras were removed, using the Bayesian classifier and the chimera.vsearch tool offered by Mothur. The filtered contigs were finally clustered into Operational Taxonomic Units (OTUs), using a cut-off value equal to 0.05 and the VSEARCH clustering method offered by Mothur. The total and individual occurrence of the reads was maintained and monitored during the whole procedure.

The statistical analysis was based on the biome file produced by Mothur and was accomplished using the R packages Phyloseq (v.1.32.0), DESeq2 (v.1.28.1) and Vegan (v.2.5-6). The variation of the microbial communities within and between samples was characterised using the alpha and beta diversity, respectively. The α-diversity evaluation was based on the rarefaction curves measuring the Shannon, Chao1, Abundance-based Coverage Estimator (ACE), and the Simpson diversity indices: Simpson Index of Diversity (1-D) and Inverted Simpson (Simpson’s Reciprocal Index, 1/D). Meanwhile, the β-diversity was evaluated by non-metric multidimensional scaling (NMDS), assessing the microbiome structure. The species abundance and field location were related based on the canonical correspondence analysis (CCA). A graphic interpretation of the main principal axes by tri-plot on the two dimensions was also obtained. All graphic representations were built using the R package ggplot2 (v.3.3.2). Venn and UpSet plots were designed using the R packages VennDiagram (v.1.7.1 and UpSetR (v.1.4.0), respectively.

### 2.4. Phylogeny-Based Functional Annotation

The PICRUSt software (v.2.3.0_b) [55] was used to predict the functional potential of the identified microbial communities. The functional prediction was based on the unique OTU sequences and the biome file produced by Mothur. The analysis returned the relative abundance of the predicted EC codes and their related pathways description. The statistical analysis and final visualisation of the results were performed using the Statistical Analysis of Metagenomic Profiles (STAMP) software (v.2.1.3) [56]. The phylogenetic analysis of the identified organisms was accomplished using Mothur and the taxonomic characterization of the sequences was based on their alignment on the recreated seed database offered by Mothur and SILVA (release 132). The taxonomy file produced by Mothur was converted to a compatible Krona tabular file (v.2.6.1) [57], which was used for the taxonomic characterisation and size overview of the identified microbial communities.

## 3. Results

### 3.1. Seasonal Shifts in Microbial Communities’ Diversity

A total of 2,531,230 reads were generated after Illumina MiSeq sequencing (Table S1). Following quality control and filtering, the obtained 440,662 sequences were aligned (based on 80% assignment bootstrap-confidence) and clustered into 438,192 OTUs (cut-off value equal to 0.03). Rank abundance curves for the top 100 OTUs showed that in summer, during the runner bean cultivation, the soils were dominated by a higher number of OTUs, indicating greater species richness in microbiome composition. Additionally, soils from autumn and summer showed higher evenness and thus, a more uniform species distribution with abundances of different species being similar (Figure S1). On the contrary, the steep drop observed in spring soils indicated an unevenness of species abundance within the population.

**Figure 2:**
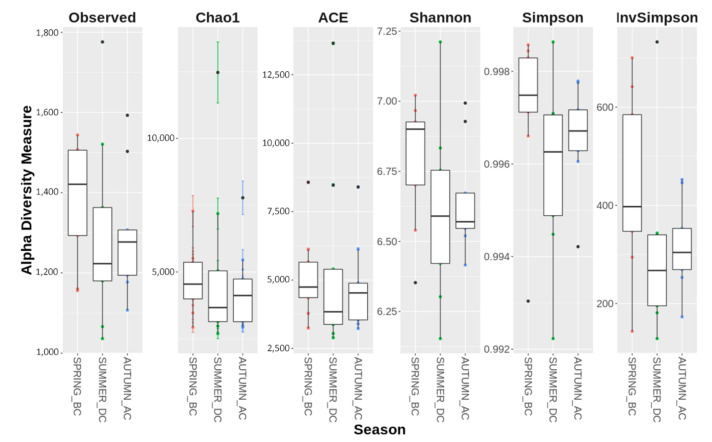
Box-plots of alpha (*α*) diversity indices. The *α*-diversity of the soil bacterial community as affected by seasonal changes and cultivation period was evaluated using the following indices: Observed richness, Chao1, ACE, Shannon, Simpson Index of Diversity and Inverted Simpson (Simpson’s Reciprocal Index). Spring_before cultivation (BC), Summer_during cultivation (DC), Autumn_after cultivation (AC) (number of sites: n = 9). Inferences were made based on the observed trends, yet no significant differences between the seasons were observed.

The quantitative shifts in bacterial community composition among the studied seasons were corroborated by differences in richness indices such as Chao1, the number of observed OTUs, and the diversity indices, Shannon and Simpson, reflecting both the evenness and species richness (Figure 2). A difference between the higher number of observed OTUs in spring soils and the lower number of observed OTUs in summer and autumn soils indicated that the runner bean possibly affected the richness of the soils during and after cultivation (Figure 2). The indices Abundance-based Coverage Estimator (ACE) and Chao1 (Abundance-based estimator), indicated comparable species richness of the microbiota in the three seasons. Moreover, a trend of higher Shannon and Simpson indices in spring were indications of greater diversity in the soil microbiome, whereas diversity was lower in summer and autumn species, but, given the higher evenness as observed based on the rank abundance curves, it could be a result of lower species richness (Figure 2).

### 3.2. Effects of Cultivation Period on the Bacterial Community Structure

**Figure 3:**
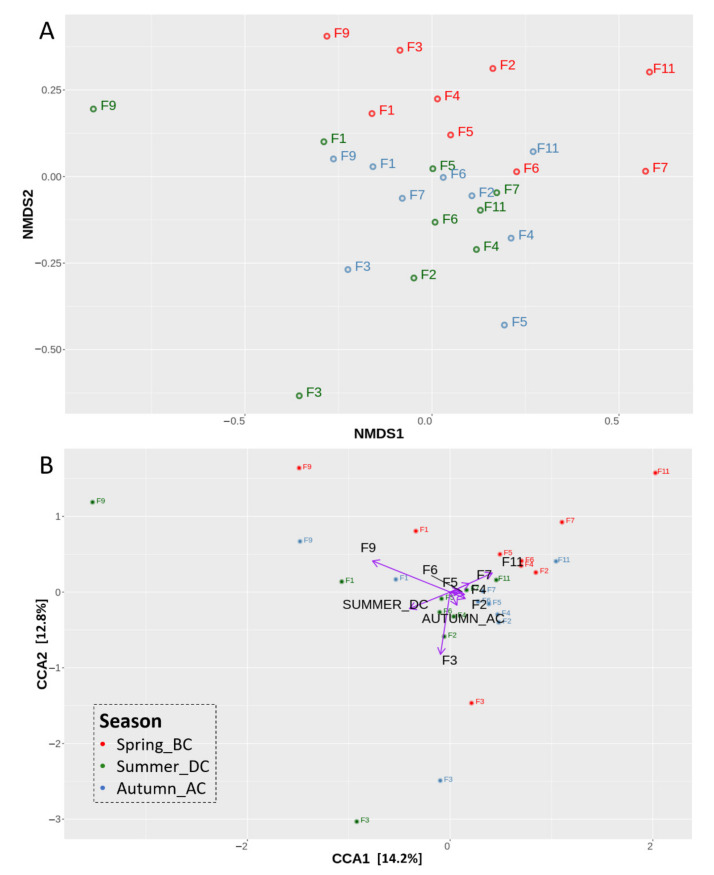
Nonmetric multidimensional scaling (NMDS) plot representing bacterial communities’ structures similarity (**A**) and Canonical correspondence analysis (CCA) of the relative variance of OTUs in the soil samples representing the effect of runner bean cultivation period on the bacterial community abundance (**B**) for nine sites (F1–F11) over three different seasons: Spring_ before cultivation (BC), Summer_during cultivation (DC), Autumn_after cultivation (AC), based on the presence/absence of runner bean crop. The weights of the most explanatory variables are represented with black font colour and purple arrows.

To investigate the differences in population structure in the soils sampled across the three seasons, the beta (β) diversity was analysed. The NMDS analysis revealed spatial separation between the spring soils (BC) and a cluster formed by the summer (DC) and autumn (AC) soil samples (Figure 3A). To further correlate bacterial communities’ composition with the different seasons and field location, Canonical Correspondence Analysis was performed (Figure 3Β). The variation explained by the seasons and field location (constrained ordination) was 40.83% and the remaining 59.17% of the variation was explained by the unconstrained ordination (Table S2). The eigenvalues of the constrained axes showed that 14.16% of the variation is explained by the CCA1 and the 12.84% by CCA2 (Table S3). The factors responsible for most of the explained variation in the bacterial community were identified as the summer season (DC), as well as the field sites F9 and F11. Field site F9 was positively correlated with DC soils, whereas site F11 showed a negative correlation (Table S4). Therefore, the bacterial community abundance during summer, especially in the fields F9 at the west side and F11 at the east side of the Mikri Prespa lake, was differently affected.

### 3.3. Effects of Cultivation Period on Different Taxonomical Levels

Soil bacterial composition for all the field sites over the three seasons, and the corresponding presence/absence of the runner bean crop, was assessed by bacterial relative abundance analysis at the phylum and class taxonomic levels. The interactive phylogenetic tree demonstrates the relative abundance (%) of phyla and classes detected in the different field sites across the three seasons Spring_BC, Summer_ DC, Autumn_ AC (Scheme S1). Altogether, 13 different phyla with OTUs representation >1% were identified across all fields and the three seasons (Table 1 and Figure 6A and Figure S2). The most abundant phyla were the Proteobacteria (~29%), Acidobacteria (~15%), Actinobacteria (~11%), and Planctomycetes (~10%), followed by Bacteriodetes (~8%) (Figure S2 and Table 1). The most affected phyla in terms of variable abundance among the different seasons were the Chloroflexi, Cyanobacteria, Patescibacteria, Firmicutes, and Nitrospirae.

Cyanobacteria were absent in BC soils and were only present in DC and AC soils (Table 1). Chloroflexi was present across all seasons with increased abundance during cultivation (DC) compared to after (AC) and before (BC) cultivation (Table 1). Patescibacteria and Nitrospirae were absent from DC soils and showed a lower abundance in AC soils compared to BC soils (Table 1). Firmicutes showed a greater abundance in DC soils, followed by AC soils, whilst BC soils showed the lowest abundance (Table 1).

**Figure 4:**
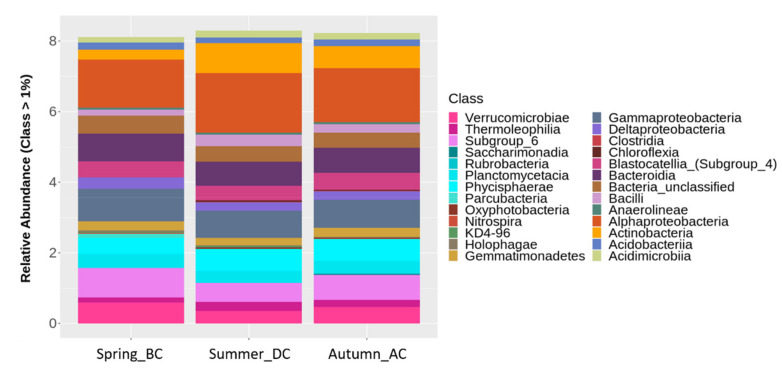
Classification of bacterial classes based on relative abundance at rate greater than 1% over three seasons: Spring_before cultivation (BC), Summer_during cultivation (DC), Autumn_after cultivation (AC) of the runner bean crop across the soil samples of nine field sites. The different classes are depicted in different colours.

**Figure 5:**
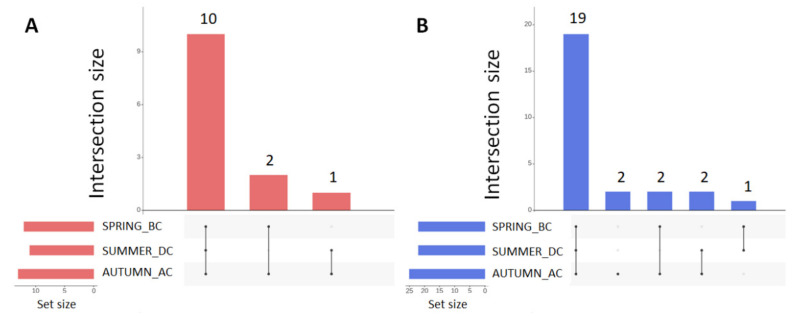
UpSet plots for grouping co-occurring variables based on their frequency at the phylum (red) and class (blue) levels. Frequencies of the detected phyla (**A**) and classes (**B**) among the different seasons (Spring_BC, Summer_DC and Autumn_AC) across all nine field sites.

Analysis of the bacterial abundance at the class taxonomic level across all fields over the three seasons, revealed that the most abundant bacterial classes, especially during the runner bean cultivation in summer, were the Alphaproteobacteria, Gammaproteobacteria, Actinobacteria, Bacteroidia and Phycisphaerae, Acidobacteria (Subgroup 6), Blastocatellia (Subgroup 4), along with Verrucomicrobiae, Planctomycetacia, and Bacilli (Table 2 and Figure 4). The runner bean cultivation affected the abundance of microbiota, with AC soils showing a greater number of classes (25 classes), compared to BC and DC soils (22 classes) (Figure 5 and Figure S3). More specifically, classes of Rubrobacteria (Actinobacteria) and Saccharimonadia (Patescibacteria) were detected solely in AC soils, whereas Nitrospira (Nitrospirae) and Parcubacteria were present mainly in soils before (BC) and after cultivation (AC) of the runner bean (Table 2 and Figure 4 and Figure S3). Additionally, Oxyphotobacteria and Clostridia were absent from BC soils and the class Holophagae was detected only in BC and DC soils (Figure 4 and Table 2).

In total, 61 genera have been identified (Figure S4; Table S5). Agronomically important genera for leguminous crops belonging to the Alphaproteobacteria and Gammaproteobacteria were detected, such as unclassified *Rhizobiales* at 1.06% detected only in BC soils, and unclassified *Burkholderiaceae* present in different abundances among the three seasons (1.73% in DC soils against 1% and 1.35% in BC and AC soils, respectively). Non-rhizobial endophytes and rhizoplane bacteria were mainly the genus *Bacillus* and unclassified *Bacillales*, which were in higher abundances during runner bean cultivation in summer (Bacilli in total: 3.53% in DC against 0.82% in BC and 1.39% in AC). Non-nodulating bacteria also include the genera *Sphingomonas* and *Massilia,* which were both detected in higher abundances in DC soils (8.5% and 1.2%, respectively) compared to spring and autumn soils. Other genera, such as the *Candidatus alysiosphaera* (1.66% only in DC soils) and the *Sphingomonas* (8.48% in DC against 6% and 7% in BC and AC soils, respectively), along with unclassified *Sphingomonadaceae* (2.53% in DC against 0.9% and 1.38% in BC and AC soils, respectively), were mainly enriched during the runner bean cultivation in summer (Figure S4; Table S5).

### 3.4. Predicted Functional Diversity of the Microbiome Present in the Different Field Sites

**Figure 6:**
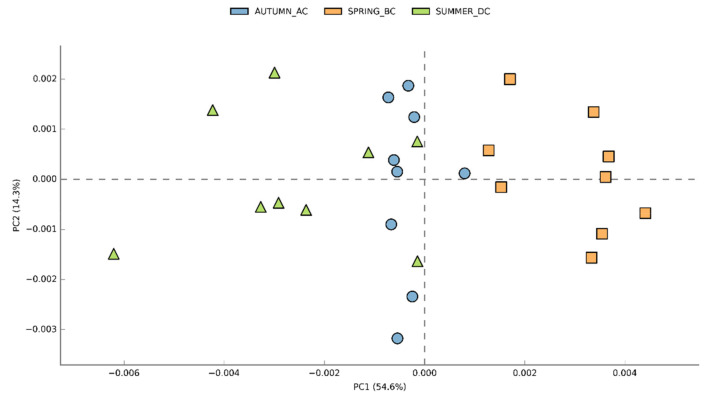
Principal Component Analysis (PCA) of the functional diversity among nine fields across three seasons: Spring_before cultivation (BC), Summer_during cultivation (DC), Autumn_after cultivation (AC). The different seasons are depicted in different colours and shapes.

**Figure 7:**
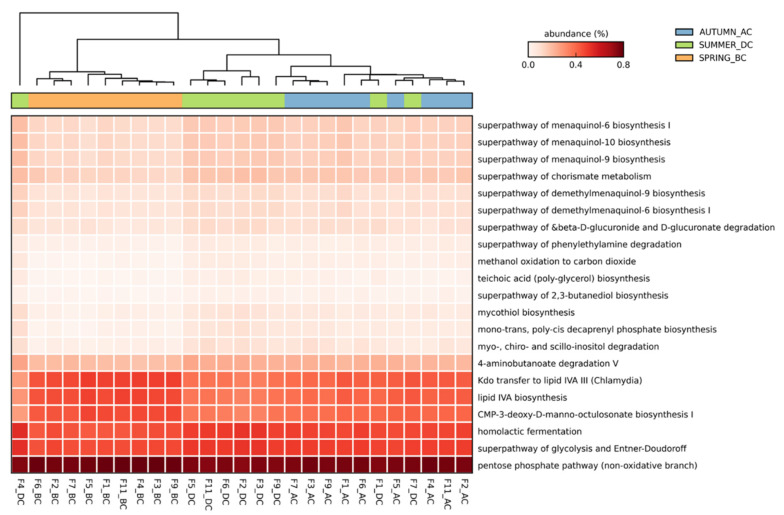
Heatmap of the predicted functional profile for nine fields across three seasons: Spring_ before cultivation (BC), Summer_during cultivation (DC), Autumn_after cultivation (AC) analysed using the STAMP software. The key shows the % relative abundances for *p*-value ≤ 0.05 and effect size > 0.7 (n = 3).

The functional diversity of the soil microbial communities was significantly different amongst the different seasons. The predicted functional profiles revealed different metabolic capacities between the microbiota present in the soil before (Spring_BC), after (Autumn_AC), and during (Summer_DC) bean cultivation. Therefore, the presence or absence of the runner bean crop, expressed as seasonal shifts, explained the 54.6% (PC1) of the functional variation, whereas a 14.3% was explained by the differences among the fields in the different seasons (Figure 6). Only 239 out of 441 pathways passed the filter of *p* < 0.05 with an effect size <0.76, and 21 pathways passed with an effect size >0.7 (Figure 7; Table S3). Prior to bean crop cultivation (BC soils), a lower abundance of sequences assigned to specific metabolic pathways was observed compared to the summer (DC) and autumn (AC) soils.

The metabolic pathways with higher effect size (>0.74) were the following four: (i) the mono-trans, poly-cis decaprenyl phosphate biosynthesis (PWY-6383), (ii) methanol oxidation to carbon dioxide (PWY-7616), (iii) chorismate metabolism (ALL-CHORISMATE-PWY), and (iv) mycothiol biosynthesis (PWY1G-0) (Figure S5). These pathways are involved in the biosynthesis of essential mycobacterial cell wall components, biosynthesis of amino acids and alcohol degradation and detoxification. In the aforementioned pathways, the % abundance was greater in DC and AC soils compared to BC soils.

Other active metabolic pathways, such as the lipopolysaccharide biosynthesis [NAGLIPASYN-PWY; lipid IVA biosynthesis and PWY-6467; Kdo transfer to lipid IVA III (Chlamydia)] and catabolism of glucose and related sugars [NONOXIPENT-PWY; pentose phosphate pathway (non-oxidative branch)], showed a greater % abundance in BC soils compared to DC and AC soils (Figure 7 and Table S6). In BC soils, the metabolic pathways with a greater abundance were involved in sugar nucleotide biosynthesis [PWY-1269; CMP-3-deoxy-D-manno-octulosonate biosynthesis I]. Interestingly, DC soils had the lowest abundance in the NAGLIPASYN pathway (Figure 7 and Table S6). The glycolysis and Entner-Doudoroff superpathway [GLYCOLYSIS-E-D], as well as teichoic acid (polyglycerol) biosynthesis [TEICHOICACID-PWY] showed a higher abundance in DC soils, followed by AC and BC soils, indicating a possible interaction of the bean crop with the soil microbiome (Figure 7 and Table S6). Additionally, the DC and AC soils showed a higher abundance in sequences assigned to metabolic pathways of 2,3-butanediol biosynthesis [PWY-6396] and phenylethylamine degradation [PWY-6071] compared to the BC soils (Figure 7 and Table S6).

Important pathways contributing to the functional potential of the soil microbiome in relation to the presence of the runner bean crop (DC and AC soils) were related to GABA degradation [4-aminobutanoate degradation V; PWY-5022], biosynthesis of menaquinones (MK), and demethylmenaquinones (DMK) [menaquinol-9 biosynthesis; PWY-5845, menaquinol-6 biosynthesis I; PWY-5850, menaquinol-10 biosynthesis; PWY-5896, demethylmenaquinol-6 biosynthesis I; PWY-5860, and demethylmenaquinol-9 biosynthesis; PWY-5862], along with lactic acid fermentation [homolactic fermentation; ANAEROFRUCAT-PWY] for converting sugars into cellular energy and lactate as a metabolic by-product (Figure 7 and Table S6). In these pathways, BC soils showed the lowest abundance of sequences. Interestingly, DC and AC soils also showed greater myo-inositol catabolism [myo-, chiro- and scillo-inositol degradation; PWY-7237] and degradation of β-glucuronides [β-D-glucuronide and D-glucuronate degradation; GLUCUROCAT-PWY], which are both possibly used as a source of carbon for growth (Table S6).

## 4. Discussion

Plant–microbe interactions are critical for plant nutrient acquisition, development, and alleviating the effects of adverse environmental conditions [58,59], whilst microbe–microbe interactions play an important role in shaping microbiota structure in plant systems [60]. In this concept, an active soil microbiota plays an important role in various soil-based ecosystem services, such as nutrient cycling, erosion control, and pest and disease regulation. The present study investigated potential shifts in microbiota diversity, the dominance of bacteria that have the role of key hub taxa, and important functional traits that may be affected by the cultivation of the runner bean in fields around the lake, Mikri Prespa, during summer, along with the before and after cultivation effects in spring and autumn, respectively. We demonstrated that the presence of runner beans differentially shaped the soil microbial community structure compared to the fallow land before and after cultivation periods, and that the effects on the soil microbiome after crop cultivation were more similar to that of summer than of spring soils.

Temporal variability in the diversity and composition of soil microbial communities has been observed especially in agricultural soils [61]. Analysis of the alpha diversity revealed variability in the bacterial community for the summer and autumn soils, possibly due to the runner bean cultivation in summer and the indirect prolonged effect on the soil in autumn. The observed similarity in species richness of the soil microbiome among the three seasons, and the higher diversity during spring, possibly indicate that during and after the cultivation period (summer and autumn, respectively) species evenness was lower and therefore the species distribution varied. Given that the mean precipitation and temperature in summer (36 mm and 21 °C) and autumn (90 mm and 2 °C) were very different, and that spring climatic conditions (59 mm and 5.8 °C) were more similar to those in autumn, we speculate that the runner bean cultivation was probably implicated in the selection of specific bacteria by recruiting or excluding specific bacterial classes or even phyla.

The microbial co-occurrence and co-exclusion patterns are important aspects for understanding the interactions orchestrating microbial community assembly [62]. In our study, Cyanobacteria and Firmicutes were positively affected by the presence of the runner bean crop along with Bacilli, with various plant growth promoting functions [63] and biocontrol activity [64] and Clostridia, which perform beneficial functions for the plants, such as atmospheric nitrogen fixation, phosphate solubilization, and the reduction of Fe^3+^ to the more readily available iron form Fe^2+^ [65]. The classes Parcubacteria and Saccharimonadia, along with *Nitrospira,* were absent from summer soils (DC), indicating that the presence of the runner bean may have negatively affected their abundance, given that both Patescibacteria abundances were either progressively increased from autumn (AC) to spring (BC) soils, or exclusively present, in the autumn soils (AC). Additionally, Gemmatimonadetes, with approximately similar abundances among the three seasons, and *Nitrospira,* that was higher in spring and absent from the DC soils, it has been shown that they can fine-tune carbon and nitrogen intakes according to their metabolic needs under heterogeneous conditions [66,67].

The presence of the runner bean crop during summer seems to be the most influential parameter responsible for most of the variation observed in the bacterial community which, based on the taxonomic profiling, is explained by the higher similarity in bacterial abundance between summer (DC) and autumn (AC) soil communities, which were clearly distinct from the spring (BC) soils. In our analysis, the identified dominant phyla were taxonomically congruent groups of bacteria belonging to Actinobacteria, Bacteroidetes, Firmicutes, and Proteobacteria, which have been shown to largely dominate the microbial assemblages associated with different plant species, as reviewed by Hacquard et al. [29], and Acidobacteria which often dominate soil bacterial communities [68,69,70]. Interestingly, we observed that the most abundant taxa varied among the seasons, and classes that prevailed during cultivation of the runner bean were different from those in spring (BC) soils. For instance, summer (DC) soils had a greater abundance of Actinobacteria (9.35%) compared to the reduction in abundance observed in autumn (AC; 6.7%) and spring soils (BC; 3.09%), which could be the effect of the runner bean crop.

Plants are critical factors influencing soil microbial community structure. Studies have shown that different plant groups, such as legumes and grass, are differentially selective for the microbial community structure and that legumes enhance the diversity of the microbiome more than grasses [71]. Herein, Gammaproteobacteria and Alphaproteobacteria prevailed in all three seasons, yet had variable responses to the presence and absence of the runner bean crop. Gammaproteobacteria (10.28%), that are known to be sensitive to low soil moisture [72] and thrive in high carbon availability [73], could show a higher abundance in spring due to the soil moisture being increased as a result of higher precipitation, whereas Alphaproteobacteria were rather enriched (18.79%) during the runner bean cultivation in summer. This further supports the evidence that consortia of beneficial microorganisms, rather than specific taxa, may drive soil ecosystem functions, due to the developed synergies [58]. Previous studies on the interactions between plant roots and microbial communities in the rhizosphere have shown that leguminous plants form symbiotic relationships with diverse bacteria belonging to Alphaproteobacteria and Betaproteobacteria [73]. In our study, non-rhizobial endophytes and rhizoplane bacteria were mainly the genus *Bacillus*, unclassified *Bacillales*, *Sphingomonas, Burkholderiaceae*, which include known nitrogen-fixing bacteria [74], and *Massilia,* a major rhizosphere and root-colonizing bacteria of many plant species [75], in higher abundances during runner bean cultivation in summer along with *Candidatus alysiosphaera*, which was solely detected during the runner bean cultivation. Research has shown that both climate and host interactions may influence root nodule-associated bacteria isolated from leguminous plants [37]. Nevertheless, the rhizobia genera identified were the unclassified *Burkholderiaceae,* present in higher abundances during summer cultivation, yet the *Rhizobiales* spp. were detected solely in the spring soils.

Studies have shown that legume-based cover crops increase soil nitrogen concentration by supporting the growth of nitrogen-fixing bacteria and thus improve the overall soil quality and enhance microbial diversity in the soil [76]. A study by Zhou et al. [71] showed that other legume species enriched *Nitrospira* in the soil microbiome, an abundant bacterium with a key role in the nitrogen cycle. Herein, *Nitrospira* was depleted from DC soils, yet was abundant in spring soils (BC) and in lower abundances in AC soils, which may indicate that other nitrogen-fixing bacteria might compensate for the absence of *Nitrospira* in the DC soils and also that the runner bean is likely incompatible with this specific phylum of nitrogen-fixing bacteria. Another important phylum, Chloroflexi, and specifically the class KD4-96 (groups 2, 3 and 4) and the genus *Roseiflexaceae* (Chloroflexia), were mainly enriched during summer (DC). Members of the phylum Chloroflexi are known to stimulate plant growth and, are involved in biocontrol [77].

In plant-soil ecosystems, Acidobacteria communities represent an important microbial guild, having, among other roles, the modulation of nutrient cycles, such as carbon, nitrogen, and sulphur [78,79]. Research on various terrestrial ecosystems indicated several subdivisions of Acidobacteria representing the keystone taxa in grasslands, forests or woodlands (subgroup 4) and plant-associated microbiota (subgroup 1, 3 and 6) [21,22,80,81]. This potentially explains the presence of different classes of Acidobacteria that were more abundant in spring soils (BC), such as subgroup 6 compared to Blastocatellia (subgroup 4), with comparable abundances in the different seasons. Additionally, the class Holophagae was also mainly present in spring (BC) and in a lower abundance in summer (DC) soils. This class has been shown to respond to the leek rhizosphere rather than in bulk soil and the rhizospheres of grass and potato [82]. Members of the Verrucomicrobia have also been shown to be present in varying plant–soil ecosystems [82,83] and herein were also observed in a higher abundance in spring (BC) and autumn (AC) soils compared to summer (DC). Therefore, the rhizosphere of the runner bean appears to have a rather negative effect towards subgroup 6, Holophagae and Verrucomicrobia. This might be explained by the oligotrophic nature of Acidobacteria and Verrucomicrobia, which are more abundant in nutrient-deprived soils [84], given that nitrogen fixing bacteria were abundant and fertilization was applied during the runner bean cultivation. Studies have shown that fertilization might change the structure of the soil microbiome by altering trophic food–web interactions [85], or that the interaction between fertilizer and the presence of legumes may affect bacterial communities’ structure [86]. We hypothesize that the shifts in community structure observed in our study may be partially explained by differences mainly in nutrient resources. Nevertheless, further investigation is required to test this hypothesis.

Soil is the source of various biological processes performed by the native microbiome, including residue decomposition, biological nitrogen fixation, nutrient cycling, and denitrification [87]. Adding to that, the abundance of sequences assigned to specific metabolic pathways was higher in summer (DC) and autumn (AC) compared to spring (BC) soils. More specifically, the metabolic pathways associated with the runner bean cultivation (DC) and the period directly after cultivation (AC) were involved in the biosynthesis of essential mycobacterial cell wall components, as well as pathways involved in carbon metabolism and detoxification. Soils during runner bean cultivation had the lowest abundance in the biosynthetic pathway of lipid-A-precursor, which is required for the outer membrane growth of Gram-negative bacteria [88]. As such, several classes of Gram-negative bacteria, including Acidobacteria, Bacteroidetes, Gemmatimonadetes, Nitrospirae, and Verrucomicrobia, showed a reduced abundance in these soils. In spring soils (BC), the metabolic pathways with a greater abundance were assigned to lipopolysaccharide biosynthesis, the sugar-nucleotide biosynthesis, as well as the catabolism of glucose and related sugars. Sugars are the most important carbon and energy source for soil microorganisms for maintaining and stimulating microbial activities in the rhizosphere, leading to the mobilization of nutrients by accelerated soil organic matter decomposition [86], which is also an aspect of fallow lands.

Important pathways contributing to the functional potential of the soil microbiome in relation to the presence of the runner bean (DC and AC soils) were related to biosynthesis of menaquinones and demethylmenaquinones, and gamma-aminobutyric acid (GABA) degradation. More specifically, the menaquinone (vitamin K_2_) biosynthesis pathway is essential in bacterial electron transport and in sensing environmental changes, such as alterations in redox state [89,90]. In the rhizosphere, the menaquinones aid in complex colony formation in *B. subtilis* [91] and have been previously shown to be important for sporulation [92]. Wicaksono et al. [93] showed that specific genes involved in menaquinone biosynthesis were predominant in Actinobacteria, and suggested an association with vitamin K_2_ biosynthesis being the main electron carrier under low oxygen concentration [94]. Herein, the higher abundance of Actinobacteria and Bacilli in DC and AC soils could be associated with the increased menaquinone biosynthesis observed in these seasons. Soil microorganisms produce GABA, which is often detected in root exudates and has a role in quorum sensing and in interbacterial and plant–bacterial interactions [95,96]. In plants, GABA is mainly involved in regulating plant development, the response to abiotic and biotic stress factors [97], as well as maintaining a carbon/nitrogen (C/N) balance, after being degraded and put through the TCA cycle [98]. The involvement of GABA in multiple processes in roots and as a signalling molecule [97], along with its impact on the root-associated bacterial communities [99], could indicate the possibility that specific soil microorganisms could use GABA as a nutrient source in the rhizosphere and thus modulate plant GABA levels. GABA metabolism genes have been previously found in *Bacillus megaterium* [100], and in our study in DC and AC soils where *Bacillus* spp. and unclassified *Bacillales* were in abundance.

During the cultivation of the runner bean (DC) in summer and later in autumn (AC), we observed significantly higher lactic acid fermentation functions contributing to the conversion of carbohydrates into cellular energy and the metabolic byproduct lactate via the Embden-Myerhof glycolytic pathway, which showed higher activity in DC soils, followed by AC and BC soils. This metabolic pathway mainly occurs in lactic acid bacteria (LAB), such as those belonging to the *Lactobacillus* species of the class Bacilli. The LAB, apart from their use in the food industry, are also used in agricultural systems to improve soils, promote plant growth, and mitigate abiotic and biotic stresses, whilst also serving as biocontrol agents and can be used as biostimulants, in biofertilisers, and bioremediation processes [101,102]. Herein, *Bacillus* and unclassified *Bacillales* species were enriched mainly in the DC soils, corroborating the significant increase in lactic acid fermentation processes, and indicating a potential association with the runner bean crop. Research has shown that the *Bacillus* species significantly promoted plant growth in other crops, such as cabbage and leak [103].

The DC and AC soils also showed greater myo-inositol catabolism and β-glucuronosides degradation, which were both used as accessible sources of carbon for promoting bacterial growth [104]. The utilization of myo-inositol is a widespread process in microbial communities and has been previously investigated, along with the superpathway of β-glucuronosides degradation, for *Bacillus subtilis* [105,106] and other Firmicutes, together with Actinobacteria, Proteobacteria, and to a lesser extent in Bacteroidetes, and specifically in plant growth promoting bacteria from the rhizosphere by forming symbiotic root nodules [104], which were also observed in higher abundances in summer and autumn soils. High myo-inositol levels were also observed in the *Pisum sativum* rhizosphere before nodule formation, as well as in ineffective nodules [107], indicating that myo-inositol might have a role in the rhizobium–legume symbiosis [108]. In the β-glucuronosides degradation process, the by-product 2-dehydro-3-deoxy-D-gluconate 6-phosphate enters the central metabolism via the Entner-Doudoroff shunt of the glycolysis pathway which, as mentioned above, also showed higher activity in DC soils, indicating a possible interaction of the bean crop with the soil microbiome. Glycolysis is one of the vital pathways in central metabolism involved in carbon metabolism [109]. The observed change in abundance of carbon metabolism pathways in soil microbiome during runner bean cultivation in summer compared to autumn and spring could potentially indicate higher CO_2_ production, which is related to the aerobic energy-yielding pathways. This could also be explained by the higher concentration of organic matter present during the runner bean cultivation. Members of the phyla Proteobacteria, Verrucomicrobia, Actinobacteria, Bacteroidetes, and Firmicutes have been shown to be important for carbon metabolism [6]. As mentioned above, Proteobacteria, Actinobacteria, and Bacteroidetes were more abundant during runner bean cultivation given that they are major cellulose decomposers. Consistently, given that Verrucomicrobia require high soil moisture [6,110], a high abundance was observed during spring, in BC soils.

## 5. Conclusions

Overall, this study was an initial attempt to understand the regulatory role of the economically important crop *Phaseolus coccineous* on the native microbial pool in the local soil environment of the lake, Mikri Prespa. Based on our analysis, the microbiome profiling from different field sites around the Mikri Prespa lake before, during, and after the runner bean cultivation has shown potential plant-beneficial and plant-growth-promoting traits predicted to be involved in nutrient availability and other functions associated with microbe–plant interactions. The specific structural shifts observed at the phylum, class, and genus taxonomic levels may potentially facilitate the management of the soil microbiome for sustainable agricultural productivity and plant protection. The seasonal shifts in relative abundance could be explained by the impact of soil organic matter based on the presence or absence of the runner bean crop, along with the balance in the processes pertaining to carbon metabolism. Soil microbiome plays a key role in increasing the availability of soil nutrients, nutrient cycling, protection of plants from biotic and abiotic stresses, as well as contributing to the regulation of plant physiology. Conservation of soil health is of paramount importance for agricultural sustainability having a central role in an agroecosystem’s productivity. This research sets the basis for soil microbiome studies in the Prespes area, which will allow for future research on the identification of beneficial bacterial taxa, especially in relation to soil health state. Based on this, agricultural management practices, involving the integration of beneficial bacteria in the soil, may arise to restore soil health and increase plant productivity and protection from pathogens. Future research should examine how these interactions are affected by the root exudates and soil chemical properties, along with other climatic factors and farming practices.

## Figures and Tables

**Table 1:** Relative abundance (%) of bacterial phyla at rate greater than 1% for Spring_ before cultivation (BC), Summer_during cultivation (DC), and Autumn_after cultivation (AC), across all field sites. The dash symbol indicates absence of the phylum from the respective season.

Phylum (%)	Season		
	Spring_BC	Summer_DC	Autumn_AC
Proteobacteria	28.96	30.04	28.49
Acidobacteria	16.91	12.41	15.29
Actinobacteria	6.26	14.37	11.25
Planctomycetes	9.50	10.76	10.71
Bacteroidetes	8.68	7.67	7.95
Verrucomicrobia	6.64	3.94	5.24
Bacteria_unclassified	5.75	4.89	4.74
Firmicutes	1.75	3.67	2.80
Gemmatimonadetes	2.83	2.30	2.79
Chloroflexi	1.21	1.68	1.21
Cyanobacteria	-	1.22	0.85
Patescibacteria	1.94	-	0.69
Nitrospirae	1.35	-	0.54
Total %	91.78	92.95	92.55

**Table 2:** Relative abundance (%) of bacterial classes at rate greater than 1% for Spring_ before cultivation (BC), Summer_during cultivation (DC), and Autumn_after cultivation (AC) across all field sites. The dash symbol indicates absence of the class from the respective season.

Phylum	Class (%)	Season		
		Spring_BC	Summer_DC	Autumn_AC
Acidobacteria	Acidobacteriia	2.27	1.77	2.13
	Blastocatellia (Subgroup_4)	4.95	4.49	5.21
	Holophagae	1.20	0.37	-
	Subgroup_6	9.29	6.02	7.95
Actinobacteria	Acidimicrobiia	1.64	2.26	2.01
	Actinobacteria	3.09	9.35	6.99
	Rubrobacteria	-	-	1.07
	Thermoleophilia	1.52	2.75	2.14
Bacteria_unclassified	Bacteria_unclassified	5.75	4.89	4.74
Bacteroidetes	Bacteroidia	8.68	7.66	7.95
Chloroflexi	Anaerolineae	0.94	0.92	0.81
	Chloroflexia	0.41	1.60	1.34
	KD4-96	0.61	0.78	0.23
Cyanobacteria	Oxyphotobacteria	-	1.22	0.85
Firmicutes	Bacilli	1.75	3.55	2.68
	Clostridia	-	1.05	1.08
Gemmatimonadetes	Gemmatimonadetes	2.83	2.30	2.79
Nitrospirae	Nitrospira	1.35	-	0.54
Patescibacteria	Parcubacteria	1.94	-	0.29
	Saccharimonadia	-	-	1.22
Planctomycetes	Phycisphaerae	5.03	7.00	6.71
	Planctomycetacia	4.47	3.75	4.00
Proteobacteria	Alphaproteobacteria	15.13	18.79	16.95
	Deltaproteobacteria	3.55	2.71	2.71
	Gammaproteobacteria	10.28	8.54	8.83
Verrucomicrobia	Verrucomicrobiae	6.64	3.94	5.24
Total %		93.32	95.71	96.46

## Data Availability

The data presented in this study are available on request from the corresponding author. The data will be publicly available with the completion of currently ongoing related studies.

## Supplementary Materials

The following supporting information can be downloaded at: www.mdpi.com/2079-7737/11/11/1595/s1, Table S1: Read counts, contigs and OTUs of the nine experimental field sites around the lake Mikri Prespa and the sampling periods in three time points (seasons/cultivation period), i.e., spring (before cultivation), summer (during cultivation) and autumn (after cultivation).; Table S2: Proportion of inertia explained by constrained and unconstrained ordination based on the canonical correspondence analysis (CCA) of the relative variance of OTUs in the soil samples representing the effect of runner bean cultivation period on the bacterial community abundance for nine sites (F1–F11) over three different seasons; Table S3: Accumulated constrained eigenvalues based on the canonical correspondence analysis (CCA) of the relative variance of OTUs in the soil samples representing the effect of runner bean cultivation period on the bacterial community abundance for nine sites (F1–F11) over three different seasons; Table S4: Biplot scores for constraining variables based on the canonical correspondence analysis (CCA) of the relative variance of OTUs in the soil samples representing the effect of runner bean cultivation period on the bacterial community abundance for nine sites (F1–F11) over three different seasons; Table S5: Relative abundance (%) of bacterial genera at rate greater than 1%, for the different seasons over all fields. Phyla and classes indicative of the genera are also shown. NA indicates absence of the genera from the respective season; Table S6: Multiple group statistics table for the predicted functional diversity in the different treatments: Autumn, Spring, and Summer, based on the abundances of sequences associated with specific metabolic pathways. The analysis was performed in the STAMP software using ANOVA with *p*-value ≤ 0.05 and effect size > 0.9; Figure S1: Rank abundance curves. Abundances of the top 100 OTUs for bacterial communities in the three seasons. Spring, summer and autumn correspond to the field conditions before, during and after cultivation of the runner bean, respectively; Figure S2: Abundance (%) of representative phyla of the bacterial community present in the different fields (F1–F11) across three different seasons (spring, summer, autumn). The different phyla are depicted with different colours; Figure S3: Abundance (%) of representative classes of the bacterial community present in the different fields (F1–F11) across three different seasons (spring, summer, autumn). The different phyla are depicted with different colours; Figure S4: Classification of bacterial genera based on relative abundance at rate greater than 1% over three seasons: Spring_before cultivation (BC), Summer_during cultivation (DC), Autumn_after cultivation (AC) of the runner bean crop across the soil samples of nine field sites. The different genera are depicted in different colours; Figure S5: Post-hoc plots for the predicted pathways demonstrating greater abundance of sequences (%) in different seasons (Spring_BC, Summer_DC and Autumn_AC) across all field sites, indicating: (i) the mean proportion of sequences within each season, (ii) the difference in mean proportions for each pair, and (iii) a *p*-value indicating whether the mean proportion is equal for a given pair. The analysis was performed in the STAMP software using ANOVA with *p*-value ≤ 0.05 and effect size > 0.75; Scheme S1: The taxonomic characterization and size overview of the identified microbial phyla and classes.
