# Supplementary material for: Seasonal Shifts in Soil Microbiome Structure Are Associated with the Cultivation of the Local Runner Bean Variety around the Lake Mikri Prespa
Source: Biology (Basel). 2022 Oct 31;11(11):1595. doi: 10.3390/biology11111595 (PMC9687114; doi:10.3390/biology11111595)
Supplement: Supplementary file 1 [file biology-11-01595-s001.zip › Scheme S1.html]

Javascript must be enabled to view this page.

members
magnitude
magnitudeUnassigned

0

438192

438192

77

64

64

64

64

13

13

13

13

9

9

9

9

9

33

33

33

33

33

203

203

203

203

203

35099

155

155

155

155

3

3

3

3

34671

15

15

15

13

13

13

2071

1597

3

1486

1

87

5

15

152

92

20

35

5

275

4

232

39

3

3

44

44

6

6

6

61

61

61

9

9

9

36

12

12

24

24

7

7

7

67

67

67

24

24

24

15

15

15

21

14

14

7

7

100

100

100

916

916

916

11

11

11

58

54

54

4

4

29

29

29

7

7

7

20

20

20

4083

1

1

5

5

219

219

6

6

6

6

4

4

4

4

1444

1444

313

313

1946

5

24

12

27

144

40

25

8

3

724

33

231

69

282

85

97

8

22

8

56

34

9

111

111

24

24

231

231

231

18

18

18

18361

47

47

13

13

18174

17

16

110

35

5

35

12

1

41

25

7

1

174

732

49

39

7

134

51

304

272

47

697

158

6

22

76

1987

732

158

5

12

17

42

5

27

356

21

380

22

71

6659

25

10

59

681

20

56

689

63

18

55

473

31

47

5

3

25

13

67

111

120

5

3

29

195

250

26

17

16

7

25

198

247

7

503

21

56

329

35

31

36

15

15

8

8

92

5

78

1

7

1

3

3

5

5

4

4

4

4

4

14

14

14

145

145

145

47

47

47

24

3

2

1

2

2

1

1

9

9

1

1

8

8

1

1

1

8

8

8

8060

237

4

1

32

2

198

60

60

10

10

16

16

939

30

188

690

6

25

42

42

4

4

6

6

35

35

4087

8

4013

22

27

4

6

7

6

6

839

839

8

8

11

11

197

197

58

58

5

5

96

25

1

3

25

28

14

51

51

219

219

7

7

27

27

3

3

11

11

970

970

4

4

14

14

49

49

49

49

5

5

5

14

14

14

7

7

7

16

16

16

12

12

12

87

87

87

10

10

10

16

16

16

22

22

22

210

155

155

155

20

20

20

32

32

32

3

3

3

3

3

3

3

38

38

38

38

4

4

4

4

8

8

8

8

7

7

7

7

117

117

117

117

117

15

15

15

15

15

46

46

46

46

46

57

57

57

57

57

36

6

6

6

6

30

30

30

30

142

142

142

142

142

3

3

3

3

3

14618

6

6

6

6

24

24

24

24

158

123

123

123

35

35

35

505

505

505

505

16

16

16

16

38

38

38

38

13

13

13

13

3306

3306

8

8

35

35

42

42

18

18

3021

3

52

26

7

53

182

139

113

4

608

22

11

37

754

61

8

12

85

146

65

6

4

17

7

9

264

178

20

41

7

13

23

10

5

17

9

3

5

5

42

42

21

21

4

4

67

67

4

4

29

29

10

10

12

12

12

12

14

14

14

14

57

57

57

57

33

33

33

33

18

18

18

18

26

26

26

26

4

4

4

4

250

250

250

250

3

3

3

3

5

5

5

5

8

4

4

4

4

4

4

5

5

5

5

4023

4023

4023

4023

12

12

12

12

3

3

3

3

19

19

19

19

48

48

48

48

4298

8

8

8

9

9

9

39

39

39

10

10

10

959

959

959

14

14

14

1598

1598

23

7

4

30

5

62

6

24

10

144

1283

13

13

13

3

3

3

436

436

436

3

3

3

7

7

7

11

11

11

123

123

123

11

11

11

293

293

293

391

391

391

33

33

33

7

7

7

4

4

4

7

7

7

153

7

7

137

137

9

9

166

166

166

349

1

1

1

346

346

346

2

2

2

9

9

9

9

12

12

12

12

6

6

3

3

3

3

1338

1338

1338

1338

14

14

7

7

7

7

7

7

4

4

4

4

4

77

77

77

77

77

119

119

119

119

119

24

3

3

3

3

9

9

9

9

6

6

6

6

6

6

6

6

24

24

24

24

24

19529

19529

689

689

689

14

14

11

3

30

30

30

371

371

371

6

6

6

3

3

3

8

8

8

22

22

22

16

16

16

13

13

13

37

37

37

12

12

12

7

7

7

11

11

11

45

45

45

12

12

12

90

90

90

10

5

5

5

5

3

3

3

116

116

116

8646

8646

180

203

20

79

31

39

26

7

28

20

36

22

172

7

77

73

170

784

13

1393

14

9

42

9

19

5

29

463

2333

184

16

4

10

33

16

21

59

19

74

66

24

17

14

30

320

159

33

3

54

51

77

5

78

35

55

35

23

293

152

7

18

216

96

13

11

11

11

7923

6933

5048

27

9

98

88

4

25

63

9

89

10

7

55

14

13

51

8

263

433

10

17

468

13

28

10

32

7

34

25

25

8

8

21

21

220

220

10

10

251

156

95

3

3

207

207

7

7

3

3

216

216

9

9

10

10

4

4

4

17

17

17

33

33

20

13

42

42

42

10

10

10

13

13

13

1323

680

674

5

1

10

10

627

627

6

6

3

3

3

14

7

7

7

7

7

7

7

7

59

59

59

59

59

22

22

22

22

22

80

80

80

80

80

19

13

13

13

8

5

6

6

6

1

4

1

36

36

36

36

36

19

19

19

19

19

47

47

47

47

47

32

32

32

32

32

31

31

31

31

31

21

21

21

21

21

33

6

6

6

6

18

18

18

18

9

9

9

9

12

12

12

12

12

4

4

4

4

4

11620

672

672

672

672

168

168

168

168

5

5

5

5

20

20

20

10

10

10

10

10

10

3

3

3

3

10628

10628

10628

22

15

16

43

5

19

83

44

18

55

9

26

34

11

7

93

740

8

21

80

3701

7

18

18

15

65

15

362

41

38

13

40

805

33

16

139

3583

20

190

16

50

41

30

23

36

36

36

36

3

3

3

3

75

75

75

75

15

15

15

15

15

12

4

4

4

4

8

8

8

8

75

75

75

75

75

34

34

34

34

34

42

42

42

42

42

38

38

38

38

38

4

4

4

4

4

3

3

3

3

3

549

427

427

427

427

64

64

64

64

58

58

58

58

8

8

8

8

8

34

34

34

34

34

11

11

11

11

11

40

40

40

40

40

11

11

11

11

11

3

3

3

3

3

12

8

8

8

8

4

4

4

4

50

50

50

50

50

175

83

83

83

83

92

92

92

92

13

13

13

13

13

115

84

84

84

84

31

31

31

31

15

15

15

15

15

271

271

271

271

271

53

53

53

53

53

25

25

25

25

25

79

79

79

79

79

282

282

282

282

282

5

5

5

5

5

561

561

561

561

561

30

30

30

30

30

2136

2133

2133

2133

2133

3

2

2

2

1

1

1

32

32

32

32

32

31

31

31

31

31

5438

2629

2629

15

15

8

8

9

9

37

37

8

8

18

18

15

15

8

8

12

12

5

5

5

5

141

141

24

24

17

17

19

19

4

4

9

9

190

190

11

11

11

3

4

4

5

5

5

5

6

6

87

87

13

13

32

32

1817

1817

12

12

17

17

7

7

62

62

106

33

33

33

39

39

39

1

1

1

30

30

30

2

2

2

1

1

1

26

1

1

1

6

6

6

16

16

16

3

3

3

3

3

3

3

12

12

12

12

192

192

192

192

6

6

6

6

20

20

20

20

78

26

26

26

39

39

39

5

5

5

1

1

1

4

4

4

3

3

3

6

6

6

6

8

8

8

8

2332

13

13

13

25

25

25

4

4

4

9

9

9

6

6

6

21

21

21

12

12

12

10

10

10

13

13

13

2

2

2

5

5

5

1483

1483

1483

7

7

7

112

112

112

12

12

12

16

16

16

99

99

99

10

10

10

23

23

23

9

9

9

9

9

9

289

289

289

66

66

66

26

26

26

39

39

39

12

12

12

10

10

10

10

6

6

6

6

4

4

4

4

65

65

65

65

65

417

347

347

347

347

70

70

70

70

54101

14

14

14

14

23

23

23

23

6

6

6

6

9934

5387

433

433

79

79

5

5

45

45

8

8

69

69

30

30

123

123

402

402

13

13

121

121

49

49

30

30

32

32

525

525

7

7

11

11

19

19

227

227

9

9

20

20

283

283

4

4

6

6

168

168

332

332

196

196

4

4

101

101

124

124

994

994

6

6

14

14

39

39

859

859

51

51

51

37

37

37

11

11

11

39

39

39

23

23

23

3

3

3

7

7

7

237

237

237

32

32

32

3039

3039

3039

141

141

141

151

151

151

26

26

26

32

32

32

17

17

17

74

74

74

7

7

7

16

16

16

83

83

83

184

184

184

24

24

24

5

5

5

10

10

10

20

20

20

201

201

201

3

3

3

13

13

13

53

53

53

8

8

8

18

18

18

18

92

92

92

92

7480

32

32

32

12

12

12

3166

15

15

15

15

15

15

99

99

38

38

1

1

121

121

29

29

17

17

20

20

6

6

2103

16

414

113

607

33

315

66

98

366

3

15

57

3

3

134

134

48

48

23

23

36

36

443

443

7

7

7

14

7

7

7

7

69

69

69

142

142

142

79

79

79

285

285

285

20

20

20

18

18

18

142

142

142

16

16

16

152

152

152

128

128

128

22

22

22

12

12

12

22

22

22

54

54

54

14

14

14

21

21

21

23

23

23

24

24

24

384

384

384

61

61

61

102

102

102

29

29

29

50

50

50

6

6

6

38

38

38

1127

1127

1127

56

56

56

9

9

9

181

169

169

12

12

29

29

29

5

5

5

5

5

5

24

24

24

207

207

207

9

9

9

13

13

13

79

79

79

77

77

77

9

9

9

22

22

22

166

166

166

4

4

4

48

48

48

266

266

266

209

209

209

209

10

10

10

10

315

315

315

315

59

17

17

17

42

42

42

104

104

104

104

109

25

25

25

37

37

37

47

47

47

21

21

21

21

84

84

84

84

34

34

34

34

11

11

11

11

119

41

41

41

78

78

78

6

6

6

6

15

15

15

15

12

9

9

9

3

3

3

22

22

22

22

9

9

9

9

35

35

35

35

83

83

83

83

22

22

22

22

3

3

3

3

47

47

47

47

18

18

18

18

65

65

65

65

16

16

16

16

18

6

6

6

12

12

12

20

10

10

10

10

10

10

1905

1905

1905

1905

58

53

53

53

5

5

5

31452

1773

1773

3

752

1

114

16

375

28

9

40

58

1

376

6085

911

829

15

2

3

10

22

30

17

17

21

21

3

3

5133

4

472

1470

29

85

104

523

123

63

25

55

7

10

11

20

169

6

10

163

169

229

134

13

127

77

156

4

869

6

30

30

30

24

24

24

133

133

133

960

960

960

3229

3229

15

6

9

94

6

2332

12

6

4

655

90

4

4

4

6

6

6

8384

3

3

212

1

8

8

187

8

5

5

4990

6

3

272

115

3

11

6

4371

58

145

79

23

56

1626

136

1

4

7

3

8

151

1316

5

5

2

2

39

39

1365

345

91

11

3

13

426

108

7

13

271

1

4

72

52

52

6

6

798

14

2

6

2

4

4

4

274

3

215

3

53

4

4

502

64

1

8

4

26

3

364

32

45

45

45

9

9

9

37

18

18

19

19

25

25

25

59

59

59

11

11

11

1275

3

2

1

53

53

186

186

9

9

6

6

760

760

36

36

216

96

21

12

3

66

5

13

6

6

7

7

7

3418

131

131

4

4

13

13

71

71

3147

32

3

3

6

491

21

14

156

2342

10

69

52

52

56

56

56

90

90

90

5

5

5

25

25

25

30

30

30

10

10

10

63

63

63

24

24

24

5

5

5

78

78

78

6

6

6

6

6

6

17

17

17

114

114

114

17

17

17

35

35

35

30

30

30

15

15

15

80

80

38

42

2863

2863

175

9

99

263

808

4

178

2

250

11

233

3

91

47

6

2

13

599

6

5

8

1

28

22

9

9

9

136

136

5

127

4

805

805

805

516

516

516

101

101

101

4

4

4

8

8

8

8

760

760

760

760

74

74

74

74

25

25

25

25

47

35

35

35

12

12

12

39

39

39

39

189

189

189

189

173

173

173

173

9

9

9

9

25

25

25

25

10

10

10

10

210

97

97

97

113

113

113

38

38

38

38

7

7

7

7

8

8

8

8

10

10

10

10

21

21

21

21

8

8

8

8

8

97

44

44

44

44

9

9

9

9

44

44

44

44

20

20

20

20

20

5

5

5

5

5

14

14

14

14

14

73

6

6

6

6

6

6

6

6

12

12

12

12

49

49

49

49

10

10

10

10

10

264

262

262

262

262

2

2

2

2

2949

2860

1390

20

4

4

10

2

257

257

5

5

767

767

2

1

1

28

28

2

1

1

309

20

101

27

2

31

126

2

8

8

8

12

12

12

7

7

7

9

9

9

37

37

37

16

16

16

767

767

767

609

609

609

5

5

5

2

2

2

2

80

1

1

1

20

20

20

55

55

55

4

4

4

7

7

7

7

36

36

36

36

36

546

546

68

68

68

478

478

478

5

5

5

5

5

35

21

21

21

21

14

14

14

14

12

12

12

12

12

72

24

24

4

4

20

20

4

4

4

4

12

12

12

12

20

20

20

20

12

12

12

12

31

31

31

31

31

153

153

153

153

153

11

11

11

11

11

1

1

1

1

1

18

18

18

18

18

3

3

3

3

3

56

56

56

56

56

7

7

7

7

7

9

9

9

9

9

13

13

13

13

13

130

130

130

130

130

12

12

12

12

12

16081

168

168

168

2

166

42

42

32

2

23

7

10

10

13984

19

19

19

13945

50

50

6

6

3

3

7

7

1931

1931

7

7

10

10

83

27

56

32

16

16

121

12

109

30

30

62

62

4

4

9

9

44

8

36

1276

7

5

57

9

24

8

44

50

2

6

28

2

29

868

133

4

52

52

9

9

36

36

15

5

5

5

13

13

37

37

1021

1021

514

63

6

57

106

22

13

1

22

7

82

4

120

5

6

32

32

592

592

3

3

47

47

42

42

6

6

45

45

2198

2198

1183

1167

16

102

102

20

20

3645

8

253

3

2

2902

6

15

3

3

12

31

9

4

14

58

3

20

3

2

17

5

5

11

256

175

175

16

16

6

6

461

196

24

1

64

39

2

24

3

94

14

8

8

8

9

3

3

3

2

1

2

2

1

1

3

3

3

35

12

12

12

23

23

23

6

6

6

6

118

118

118

118

1642

29

29

29

3

3

3

5

5

5

3

3

3

3

3

3

18

18

18

4

4

4

1577

173

173

74

1

24

13

15

3

8

2

1

4

3

8

8

1

1

2

1

1

11

11

42

17

19

6

71

8

16

47

2

2

24

4

4

16

346

1

2

4

4

4

6

78

18

58

14

56

85

3

13

203

32

84

1

37

2

2

2

7

6

4

2

3

4

5

1

1

7

3

3

3

3

3

6

6

297

5

13

200

32

38

9

115

115

19

1

4

14

25

25

152

3

18

20

1

2

18

1

67

22

9

9

9

9

10

5

5

5

5

5

5

8

8

8

8

45

45

45

45

14

14

14

14

27

27

27

27

27

28

28

28

28

28

12

12

12

12

12

11

11

11

11

11

10

10

10

10

10

1532

551

551

551

551

7

7

7

7

6

6

6

6

389

389

389

389

449

449

9

9

69

69

137

137

11

11

92

92

48

48

20

20

4

4

10

10

22

22

27

27

8

8

8

8

3

3

3

3

83

83

83

83

12

12

12

12

21

21

21

21

3

3

3

3

48

48

48

48

48

73

73

73

73

73

254

37

37

37

37

217

217

217

217

56

56

56

56

56

70

25

25

25

25

45

45

45

45

11

11

11

11

11

3

3

3

3

3

9

9

9

9

9

14

14

14

14

14

68

68

68

68

68

36

36

36

36

36

28

28

28

28

28

92

92

92

92

92

19

19

19

19

19

149

77

77

77

77

72

72

72

72

212

212

212

212

212

24

24

24

24

24

28

28

28

28

28

509

509

509

509

509

155

155

155

155

155

18

18

18

18

18

247

247

247

247

247

30

20

20

20

20

10

10

10

10

70

70

70

70

70

119

119

119

119

119

14

7

7

7

7

7

7

7

7

5

5

5

5

5

11

11

11

11

11

46

46

46

46

46

20

16

16

16

16

4

4

4

4

20

20

20

20

20

19

19

19

19

19

77

77

77

77

77

21

7

7

7

7

7

7

7

7

7

7

7

7

48

48

48

48

48

22

22

22

22

22

9

9

9

9

9

205

205

205

124

124

3

3

11

11

67

67

63

63

63

63

32

1

30

13

13

13

13

13

92

92

92

92

92

13

13

13

13

13

8

8

8

8

8

7

7

7

7

7

145

145

145

145

145

81

81

81

81

81

598

598

32

32

32

566

566

566

17

17

17

17

17

44

44

44

23

23

3

3

18

18

35

35

35

35

35

138

138

138

138

138

11

11

11

11

11

70

70

70

70

70

22

22

22

22

22

73

73

73

73

73

26

26

26

26

26

12

12

12

12

12

12

6

6

6

6

6

6

6

6

76

76

76

4

4

4

4

42

42

26

26

11

11

11

11

11

77

77

77

77

77

193

131

131

131

131

62

62

62

62

128297

20

10

10

10

10

10

10

13

13

13

13

44

44

44

44

5

5

5

5

40451

1472

1472

1472

8301

8

8

13

13

105

105

761

4

326

6

19

14

4

3

2

47

43

11

282

8

8

272

272

5

5

7012

3

12

5

575

76

941

40

28

515

15

2

8

4

33

2224

16

25

19

50

456

1122

159

7

78

58

94

101

5

111

138

92

4

4

58

58

55

55

932

16

10

1

5

8

8

908

908

2

2

2

2

2

2

34

34

34

7

7

7

10

10

10

204

201

199

2

3

3

10

10

1

3

6

7

7

7

169

169

2

1

23

50

78

11

2

2

3

3

3

2

2

2

316

316

1

32

223

30

15

15

2481

2481

1796

356

45

90

81

3

34

34

4

4

34

18

18

18

144

144

1

1

142

15

15

15

296

38

10

2

26

257

235

1

5

16

1

1

28

28

2

26

4

4

4

2

2

2

2

2

2

3

3

3

19

19

19

17

17

17

4

4

4

22

22

22

10

10

10

28

28

28

25

25

25

3

3

3

643

643

643

17

17

17

1

1

1

23311

3245

3245

9

9

22

22

5

5

1132

1132

116

116

11

11

9

9

957

957

42

42

12

12

18

18

134

134

20

20

3

1

2

15

15

12

12

31

31

8

8

11838

7

139

102

42

4

3

143

71

44

3

1

6

9

7

17

24

182

10

76

67

68

1

130

354

20

6

440

1272

1

66

4

8

21

3

7

5

4

93

69

28

2

18

68

17

5

25

42

31

17

1

41

73

1

5

1174

9

3

8

1

8

23

2

100

43

3

15

2520

1

11

3

10

895

379

3

49

114

26

90

201

164

5

45

4

7

5

33

26

10

6

341

1

152

3

322

4

5

37

13

3

781

5

88

31

2

43

15

93

4675

174

14

139

1953

24

1788

583

14

14

13

13

12

12

7

7

18

18

12

12

66

66

156

156

3

3

36

36

6

6

117

1

22

1

8

80

3

2

64

64

23

23

435

96

7

7

18

16

1

10

27

10

180

60

3

15

15

1

1

1

9

9

9

1852

1852

6

1

1845

5

5

5

5

5

5

3

3

3

4

4

4

2

2

2

6

6

6

4

4

4

4

729

729

729

729

73855

9

9

9

101

101

101

3

3

3

23

23

23

13

13

13

160

153

153

6

6

1

1

246

246

246

53

53

53

21

21

21

5

5

5

41

41

41

2

2

2

25

25

25

10

10

10

75

75

75

17650

5

5

540

10

54

261

142

40

33

15

3

12

113

113

19

19

26

26

18

18

33

33

4

4

40

20

20

10

10

124

124

264

264

90

90

28

28

970

402

4

237

109

2

17

12

187

11

11

108

108

2573

13

1

1

5

9

2

642

24

72

6

1190

23

1

37

12

4

123

358

25

6

11

7

1

157

157

138

138

26

26

1

1

4186

128

6

4

2031

1253

76

34

11

22

1

13

65

3

430

6

103

16

16

871

871

918

918

219

219

969

17

914

22

16

31

31

22

22

5

5

20

20

37

37

67

67

3

3

3

3

19

19

8

8

11

11

4

4

3995

75

57

327

135

279

20

36

23

1

69

7

1

79

144

1943

1

257

3

54

171

94

184

8

21

6

40

40

70

70

106

106

16

16

634

25

609

18

18

49

49

294

294

294

4

4

4

4

4

4

1307

1307

1307

4

4

4

82

82

82

4

4

4

33

33

33

12

12

12

12

12

12

11

11

11

32

32

32

72

72

72

96

96

96

5

5

5

3

3

3

2

2

2

6

6

6

6

6

6

142

142

142

7

7

7

25

25

25

7

7

7

14

14

14

11

11

11

30

30

3

12

5

10

12

12

12

25

25

25

13

1

1

8

5

3

4

4

15

15

15

596

596

588

8

6398

6398

6398

2634

2362

62

9

368

70

7

368

33

79

79

20

414

66

3

784

265

97

1

167

5

5

2

2

11

11

11

408

408

408

788

788

41

5

42

16

34

14

545

8

4

3

32

43

1

39798

39798

199

10

81

5301

113

120

3

20

24

359

21

2

501

6

92

7

70

9

119

126

42

27

10

350

2488

116

3

5

100

10838

30

142

24

549

187

18

11

50

1

55

3769

1166

50

10345

12

27

3

143

1

144

10

140

18

741

20

23

110

4

11

155

212

184

265

16

13

13

13

41

41

41

54

54

54

210

210

210

19

19

19

7

7

7

581

581

581

6

1

1

5

5

6

6

6

51

51

51

1297

2

2

1290

4

391

11

7

9

7

2

6

21

45

114

5

8

81

350

37

66

23

17

5

79

1

1

5

5

33

3

3

6

6

4

4

20

20

177

177

177

5

5

5

5

16

16

16

16

6

6

6

6

15

5

5

5

10

10

10

7

7

7

7

8

8

8

8

16

8

8

8

8

8

8

12950

21

21

21

5

5

5

722

221

10

178

21

12

501

234

267

9

9

9

4

4

4

463

463

463

6

6

4

2

7

7

7

29

29

29

30

30

30

7

7

7

61

61

61

16

16

16

45

45

45

332

328

328

4

4

199

199

2

197

13

13

13

37

37

37

3

3

3

6

6

6

5

5

5

20

20

7

9

4

6

6

6

5

5

5

52

12

12

4

4

4

4

1

1

28

28

3

3

10278

1002

1002

271

14

12

170

72

3

18

18

25

25

78

73

5

23

23

29

29

17

17

2308

2308

20

20

3

3

10

5

5

23

23

68

68

7

7

15

15

9

9

21

21

27

27

106

106

6

3

3

64

64

21

21

656

11

15

1

4

127

54

9

70

24

23

43

136

9

46

84

164

164

20

20

68

68

10

10

20

20

22

22

5

5

34

34

36

36

9

9

9

9

12

12

74

74

12

12

18

18

1

1

53

53

3

3

20

20

13

13

8

8

49

49

156

156

40

40

16

16

20

20

10

10

83

83

68

68

4

4

30

30

778

763

15

5

5

63

63

10

10

103

103

5

5

1396

1396

17

17

81

81

138

138

33

33

56

56

6

6

40

40

21

21

515

167

6

15

15

83

127

97

5

23

23

75

75

733

12

31

29

7

31

16

42

67

6

5

260

4

41

28

5

24

49

25

51

72

72

43

43

9

9

51

51

37

37

54

54

543

543

543

12

12

12

14

14

14

15

15

15

15

27

27

27

27

21

3

3

3

18

18

18

6

3

3

3

3

3

3

5

5

5

5

51

51

51

51

7

7

7

7

8

8

8

8

7

7

7

7

6

6

6

6

15

15

15

15

15

15

15

15

15

15

195

195

195

195

195

12

12

12

12

12

19

19

19

19

19

10

10

10

10

10

7

7

7

7

7

102

102

102

102

102

28

28

28

28

28

27

27

27

27

27

6

6

6

6

6

38

38

38

38

38

7

7

7

7

7

16

16

16

16

16

20

20

20

20

20

11

11

11

11

11

18

18

18

18

18

10

10

10

10

10

16

8

8

8

8

8

8

8

8

11

11

11

11

11

91

91

91

91

91

8

8

8

8

8

15

15

15

15

15

5

5

5

5

5

87

87

87

87

87

14

14

14

14

14

1

1

1

1

1

10

10

10

10

10

8

8

8

8

8

15

15

15

15

15

85

85

85

85

85

15

10

10

10

10

5

5

5

5

25

25

25

25

25

258

258

258

258

258

19185

19185

19185

19185

19185

33

33

33

33

33

18

18

18

18

18

6

6

6

6

6

155

139

139

139

139

16

16

16

16

139

139

139

139

139

53

53

53

53

53

51

9

9

9

9

42

42

42

42

39

39

39

39

39

46171

3

3

3

3

30

30

30

30

414

414

414

414

13

13

13

13

1

1

1

1

21

21

21

21

27193

26328

26328

26328

588

588

1

260

4

4

15

12

15

192

85

35

35

35

21

21

21

78

78

78

31

31

31

5

5

5

1

1

1

106

106

106

12

12

12

12

180

180

180

180

9

3

3

3

6

6

6

13

13

13

13

9

9

9

9

5

5

5

5

10

10

10

10

20

20

20

20

10

5

5

5

5

5

5

6

6

6

6

3

3

3

3

11

11

11

11

18018

32

32

32

14

14

14

2746

2746

2746

6

6

6

24

24

24

89

89

63

26

3894

3894

40

94

30

10

102

664

7

7

6

8

6

13

8

54

23

32

6

19

54

5

13

7

36

15

24

5

68

13

54

24

92

9

21

10

35

1420

739

9

12

30

57

13

12

12

12

6

6

6

28

28

28

18

18

18

75

75

75

10

10

10

41

41

41

31

31

31

12

12

12

9

9

9

65

65

65

11

11

11

28

28

28

65

65

65

66

66

55

11

27

27

27

41

41

41

21

21

21

13

13

13

32

32

32

49

49

49

20

20

15

5

85

85

85

20

20

10

10

2365

15

15

1102

1102

25

25

252

252

5

5

116

116

427

9

2

20

68

267

30

12

13

6

25

25

4

4

20

20

5

5

81

81

7

7

10

10

13

13

10

10

178

178

12

12

31

31

11

11

16

16

17

17

17

43

43

43

24

24

24

32

32

32

35

35

10

20

5

61

61

61

11

11

11

133

133

133

49

49

24

25

160

160

160

30

30

9

21

9

9

9

55

55

55

18

18

18

10

10

10

23

23

13

10

79

79

79

37

37

37

13

13

13

32

32

24

8

4

4

4

96

96

96

7

7

7

17

17

17

17

17

17

40

40

32

8

14

14

14

77

77

77

33

33

33

84

84

84

67

67

67

16

16

16

11

11

11

32

32

32

145

145

59

69

17

199

199

45

154

17

17

17

60

60

60

36

36

36

43

43

43

235

235

216

19

146

146

134

12

9

9

9

2253

2253

12

16

5

93

73

353

14

41

1011

14

5

6

15

21

9

120

11

111

280

21

8

10

4

10

10

10

10

10

10

23

23

23

7

7

7

21

21

21

18

18

18

2665

2665

6

33

15

4

11

5

8

18

19

8

30

5

32

15

1783

10

14

36

586

27

10

10

10

84

84

56

28

35

35

35

7

7

7

20

20

20

86

86

86

45

45

29

16

17

17

17

40

40

40

66

66

66

11

11

11

60

60

60

91

91

91

11

11

11

28

28

21

7

110

110

51

59

50

50

50

9

9

9

26

26

26

26

10

10

10

10

8

8

8

8

7

7

7

7

6

6

6

6

5

5

5

5

128

128

128

128

19

19

19

19

19

14

14

14

14

14

54

54

54

54

54

34

34

34

34

34

37

37

37

37

37

73

73

73

73

73

4

4

4

4

4

21

21

21

21

21

14

14

14

14

14

10

5

5

5

5

5

5

5

5

15

5

5

5

5

10

10

10

10

12

12

12

12

12

10

10

10

10

10

96

10

10

10

10

13

13

13

13

73

73

73

73

15

15

15

15

15

12

12

12

12

12

8

8

8

8

8

24

8

8

8

8

16

16

16

16

33

3

3

3

3

30

30

30

30

70

67

65

65

65

2

2

2

3

3

3

3

82

82

82

82

82

20

20

20

20

20

25

25

25

25

25

8

8

8

8

8

36

3

3

3

3

33

33

33

33

12

12

12

12

12

68000

886

886

886

886

14

14

14

14

9

9

9

9

53

53

53

53

2

2

2

2

33144

11

11

11

70

70

70

136

136

136

13

13

13

101

101

101

209

209

209

252

252

252

821

821

821

41

41

41

748

748

748

74

74

74

12

12

12

201

201

201

78

78

78

15

15

15

60

60

60

184

184

184

83

83

83

25602

25602

25602

347

347

347

112

112

112

257

257

257

29

29

29

266

266

266

62

62

62

289

289

289

1210

1210

1210

82

82

82

48

48

48

7

7

7

74

74

74

33

33

33

39

39

39

5

5

5

182

182

182

11

11

11

58

58

58

28

28

28

19

19

19

93

93

93

149

149

149

88

88

88

11

11

11

6

6

3

3

111

111

111

30

30

30

130

130

130

330

330

330

14

14

14

6

6

6

43

43

43

264

264

152

103

9

708

708

708

708

20426

121

121

121

9007

9007

9

73

158

7

53

455

69

55

46

1832

118

25

286

91

71

64

5

6

347

3898

6

582

34

30

40

67

363

200

17

5

5

5

9682

9682

9682

6

6

6

12

12

12

1561

1561

1561

19

19

19

13

13

13

18

18

18

18

96

96

96

96

11

11

11

11

4

4

4

4

5

5

5

5

30

30

30

30

2561

2561

2561

2561

19

19

19

19

31

31

31

31

6

6

6

6

3

3

3

3

5

5

5

5

552

552

552

552

8450

7444

7444

18

15

39

40

12

4

98

18

93

1368

143

21

98

1

25

1

22

27

30

5366

5

1

1

1

91

91

91

914

8

8

135

135

45

45

15

15

86

86

39

3

8

10

1

11

6

68

68

46

46

54

54

18

18

237

237

9

9

96

96

15

15

3

3

34

34

6

6

12

12

12

12

56

56

56

56

18

18

18

18

673

673

673

673

208

208

208

208
