# Supplementary material for: Seasonal Shifts in Soil Microbiome Structure Are Associated with the Cultivation of the Local Runner Bean Variety around the Lake Mikri Prespa
Source: Biology (Basel). 2022 Oct 31;11(11):1595. doi: 10.3390/biology11111595 (PMC9687114; doi:10.3390/biology11111595)
Supplement: Supplementary file 1 [file biology-11-01595-s001.zip › Supplementary material.pdf]

Supplementary Materials

Seasonal Shifts in Soil Microbiome Structure Are Associated with the Cultivation of the Local Runner Bean Variety around the Lake Mikri Prespa

Evangelia Stavridou [1], Ioanna Karamichali [1], Georgios Lagiotis [1], Elena Patsea [2], Maslin Osathanunkul [3,4] and Panagiotis Madesis [1,5,*]

[1] Institute of Applied Biosciences, Centre for Research and Technology, 57001 Thessaloniki, Greece
[2] A.S. "PELEKANOS", Epar.Od. Lemou-Vronterou, 53077 Prespes, Greece; epatsea90@gmail.com
[3] Department of Biology, Faculty of Science, Chiang Mai University, Chiang Mai 50200, Thailand; omaslin@gmail.com
[4] Research Centre on Bioresources for Agriculture, Industry and Medicine, Chiang Mai University, Chiang Mai 50200, Thailand
[5] Laboratory of Molecular Biology of Plants, School of Agricultural Sciences, University of Thessaly, 38446 Volos, Greece
* Correspondence: pmadesis@uth.gr

Table S1. Read counts, contigs and OTUs of the nine experimental field sites around the lake Mikri Prespa and the sampling periods in three time points (seasons/cultivation period), i.e. spring (before cultivation), summer (during cultivation) and autumn (after cultivation).

Field	Field_Repeat	Season	Season_repeat	Sample	Reads	Contigs	OTUs
F1	F1_1	Summer	Summer_1	F1_1_S1_R1_001	184986	48415	48305
				F1_1_S1_R2_001	184986		
	F1_2	Autumn	Autumn_1	F1_2_S12_R1_001	37473	14451	14337
				F1_2_S12_R2_001	37473		
	F1_3	Spring	Spring_1	F1_3_S29_R1_001	28477	10351	10336
				F1_3_S29_R2_001	28477		
F2	F2_1	Summer	Summer_2	F2_1_S2_R1_001	39797	11279	11186
				F2_1_S2_R2_001	39797		
	F2_2	Autumn	Autumn_2	F2_2_S13_R1_001	37216	16716	16593
				F2_2_S13_R2_001	37216		
	F2_3	Spring	Spring_2	F2_3_S25_R1_001	26700	9791	9744
				F2_3_S25_R2_001	26700		
F3	F3_1	Summer	Summer_3	F3_1_S3_R1_001	48050	11085	11041
				F3_1_S3_R2_001	48050		
	F3_2	Autumn	Autumn_3	F3_2_S14_R1_001	31503	8332	8316
				F3_2_S14_R2_001	31503		
	F3_3	Spring	Spring_3	F3_3_S28_R1_001	31961	15026	14983
				F3_3_S28_R2_001	31961		
F4	F4_1	Summer	Summer_4	F4_1_S4_R1_001	59859	25670	24830
				F4_1_S4_R2_001	59859		
	F4_2	Autumn	Autumn_4	F4_2_S15_R1_001	26200	8212	8176
				F4_2_S15_R2_001	26200		
	F4_3	Spring	Spring_4	F4_3_S27_R1_001	35632	17124	17099
				F4_3_S27_R2_001	35632		
F5	F5_1	Summer	Summer_5	F5_1_S5_R1_001	107034	47076	46852
				F5_1_S5_R2_001	107034		
	F5_2	Autumn	Autumn_5	F5_2_S16_R1_001	31444	6457	6436
				F5_2_S16_R2_001	31444		
	F5_3	Spring	Spring_5	F5_3_S24_R1_001	24012	9965	9946
				F5_3_S24_R2_001	24012		
F6	F6_1	Summer	Summer_6	F6_1_S6_R1_001	35271	13217	13091
				F6_1_S6_R2_001	35271		
	F6_2	Autumn	Autumn_6	F6_2_S17_R1_001	33399	15732	15663
				F6_2_S17_R2_001	33399		
	F6_3	Spring	Spring_6	F6_3_S23_R1_001	43542	17588	17544
				F6_3_S23_R2_001	43542		
F7	F7_1	Summer	Summer_7	F7_1_S7_R1_001	36101	10719	10660
				F7_1_S7_R2_001	36101		
	F7_2	Autumn	Autumn_7	F7_2_S18_R1_001	31349	10390	10343
				F7_2_S18_R2_001	31349		
	F7_3	Spring	Spring_7	F7_3_S26_R1_001	27167	6797	6777
				F7_3_S26_R2_001	27167		
F9	F9_1	Summer	Summer_8	F9_1_S9_R1_001	102016	20412	20365
				F9_1_S9_R2_001	102016		
	F9_2	Autumn	Autumn_8	F9_2_S20_R1_001	24972	7166	7122
				F9_2_S20_R2_001	24972		
	F9_3	Spring	Spring_8	F9_3_S31_R1_001	27536	8134	8122
				F9_3_S31_R2_001	27536		
F11	F11_1	Summer	Summer_9	F11_1_S11_R1_001	97373	48014	47830
				F11_1_S11_R2_001	97373		
	F11_2	Autumn	Autumn_9	F11_2_S22_R1_001	27807	10509	10491
				F11_2_S22_R2_001	27807		
	F11_3	Spring	Spring_9	F11_3_S30_R1_001	28738	12034	12004
				F11_3_S30_R2_001	28738		
**Total**					2531230	440662	438192

Table S2. Proportion of inertia explained by constrained and unconstrained ordination based on the canonical correspondence analysis (CCA) of the relative variance of OTUs in the soil samples representing the effect of runner bean cultivation period on the bacterial community abundance for nine sites (F1-F11) over three different seasons.

	**Inertia**	**Proportion**
Total	7.5983	1
Constrained	3.1021	0.4083
Unconstrained	4.4962	0.5917

Table S3. Accumulated constrained eigenvalues based on the canonical correspondence analysis (CCA) of the relative variance of OTUs in the soil samples representing the effect of runner bean cultivation period on the bacterial community abundance for nine sites (F1-F11) over three different seasons.

**Importance of components**	**CCA1**	**CCA2**	**CCA3**	**CCA4**	**CCA5**	**CCA6**	**CCA7**	**CCA8**	**CCA9**	**CCA10**
Eigenvalue	0.4391	0.3982	0.3621	0.3193	0.3117	0.2916	0.28124	0.2562	0.22977	0.21286
Proportion Explained	**0.1416**	**0.1284**	0.1167	0.1029	0.1005	0.094	0.09066	0.0826	0.07407	0.06862
Cumulative Proportion	0.1416	0.2699	0.3866	0.4896	0.5901	0.6841	0.77471	0.8573	0.93138	1

Table S4. Biplot scores for constraining variables based on the canonical correspondence analysis (CCA) of the relative variance of OTUs in the soil samples representing the effect of runner bean cultivation period on the bacterial community abundance for nine sites (F1-F11) over three different seasons.

Variables	CCA1	CCA2	CCA3	CCA4	CCA5	CCA6
F11	0.4152	0.252321	0.2192	-0.57993	0.53559	-0.03167
F2	0.1493	-0.085771	-0.162	-0.35022	-0.76966	0.336724
F3	-0.095	-0.823502	0.5408	0.09075	0.0855	-0.03669
F4	0.1418	-0.031835	-0.1672	-0.06591	-0.00521	-0.00903
F5	0.093	0.03013	-0.2539	0.3827	0.18293	0.361257
F6	0.1033	0.002217	-0.21	-0.07168	0.04216	-0.07322
F7	0.1873	0.113367	-0.141	0.38648	-0.1745	-0.80536
F9	-0.766	0.412016	0.241	-0.06224	0.01566	0.003764
Summer_DC	-0.402	-0.218706	-0.3157	-0.49499	-0.02392	-0.36332
Autumn_AC	0.0677	-0.173841	-0.3759	0.2423	0.3428	0.269503

Table S5. Relative abundance (%) of bacterial genera at rate greater than 1%, for the different seasons over all fields. Phyla and classes indicative of the genera are also shown. NA indicates absence of the genera from the respective season.

Phylum	Class	Genus (%)	Seasons		
			Spring_BC	Summer_DC	Autumn_AC
Acidobacteria	Acidobacteriia	*Acidobacteriales_unclassified*	1.4328557	NA	NA
		Bryobacter	0.469525711	1.178240744	1.550811822
		Solibacteraceae_(Subgroup_3)_unclassified	2.2742419	NA	NA
	Blastocatellia_(Subgroup_4)	*Blastocatellaceae_unclassified*	0.6555462	1.144294478	1.1448426
		RB41	2.597913011	1.9050397	2.498550822
		uncultured	NA	NA	1.0885519
	Holophagae	*Subgroup_7_ge*	1.202135067	0.372107567	NA
	Subgroup_6	*Subgroup_6_ge*	8.547790467	5.293898278	7.136563711
		Subgroup_6_unclassified	0.503095967	0.889362933	1.199608733
	Acidimicrobiia	*Acidimicrobiia_unclassified*	NA	0.558073625	0.883348325
	Actinobacteria	*Frankiales_unclassified*	NA	1.0269835	NA
		Geodermatophilaceae_unclassified	NA	1.7762136	NA
		Intrasporangiaceae_unclassified	NA	1.0727695	NA
		Micrococcaceae_unclassified	NA	1.463790517	0.978333583
		Nocardioidaceae_unclassified	NA	0.947695633	0.389957767
		Nocardioides	NA	1.5626259	NA
		Streptomyces	NA	0.55104605	0.92447905
	Rubrobacteria	*Rubrobacter*	NA	NA	1.0671689
	Thermoleophilia	*Solirubrobacter*	NA	1.00349405	0.2892812
		uncultured_ge	1.2412529	NA	NA
Bacteria_unclassified	Bacteria_unclassified	*Bacteria_unclassified*	5.748871144	4.894200233	4.737870267
Bacteroidetes	Bacteroidia	*Chitinophaga*	NA	NA	1.1021947
		Chitinophagaceae_unclassified	2.4928394	3.374419889	3.087625667
		Cytophagales_unclassified	NA	0.8396759	1.37458715
		Flavobacterium	1.331516275	NA	NA
		Pedobacter	NA	NA	1.2568887
		uncultured	1.328241857	0.251832629	0.725857771
Chloroflexi	Anaerolineae	*uncultured*	NA	NA	1.053075
	Chloroflexia	*Roseiflexaceae_unclassified*	NA	0.5920258	0.51075785
	KD4-96	*KD4-96_ge*	0.6135054	0.78112056	0.22607022
Cyanobacteria	Oxyphotobacteria	*Leptolyngbya_EcFYyyy-00*	NA	1.4740234	NA
		Nostocales_unclassified	NA	NA	1.5972658
Firmicutes	Bacilli	*Bacillales_unclassified*	NA	1.5074883	NA
		Bacillus	0.8187228	2.033904444	1.392081489
Gemmatimonadetes	Gemmatimonadetes	*Gemmatimonas*	0.711409657	1.244670086	1.218726771
		uncultured	1.620751744	0.127661044	0.911054578
Nitrospirae	Nitrospira	*Nitrospira*	1.3455842	NA	0.53511305
Patescibacteria	Parcubacteria	*Parcubacteria_unclassified*	1.771971725	NA	0.253118775
	Saccharimonadia	*Saccharimonadales_ge*	NA	NA	1.16072985
Planctomycetes	Phycisphaerae	*WD2101_soil_group_ge*	4.742002744	6.883394511	6.554805656
	Planctomycetacia	*Pirellulaceae_unclassified*	1.269961283	0.174318683	0.51744505
		Planctomycetacia_unclassified	1.387598571	0.197818386	0.3099332
Proteobacteria	Alphaproteobacteria	*Alphaproteobacteria_unclassified*	1.578354611	1.688417689	1.811244078
		Candidatus_Alysiosphaera	NA	1.6602836	NA
		Rhizobiales_unclassified	1.0612027	NA	NA
		Sphingomonadaceae_unclassified	0.924932175	2.534326663	1.383436038
		Sphingomonas	6.0517973	8.487490844	7.084360667
		uncultured	1.23446204	NA	0.513555
	Deltaproteobacteria	*Myxococcales_unclassified*	1.193315825	0.28602995	NA
	Gammaproteobacteria	*Burkholderiaceae_unclassified*	1.044504022	1.731195511	1.3471827
		Ellin6067	NA	NA	1.1152416
		Gammaproteobacteria_unclassified	1.2435361	NA	NA
		Massilia	0.3359363	1.1979706	NA
		SC-I-84_ge	0.47205594	0.41179532	0.99438146
		Stenotrophomonas	NA	NA	1.0245284
		Steroidobacter	1.1327259	NA	NA
		Xanthomonadaceae_unclassified	1.1113216	1.152154125	0.664250475
Verrucomicrobia	Verrucomicrobiae	*Candidatus_Udaeobacter*	2.288903	1.164951038	1.456827775
		Pedosphaeraceae_ge	0.47718476	0.22138836	0.44101572
		Pedosphaeraceae_unclassified	1.333414025	NA	0.2859594
		Verrucomicrobiae_unclassified	1.028037029	0.183596886	0.722070914
		**SUM**	**66.61901705**	**65.84179053**	**66.52075418**

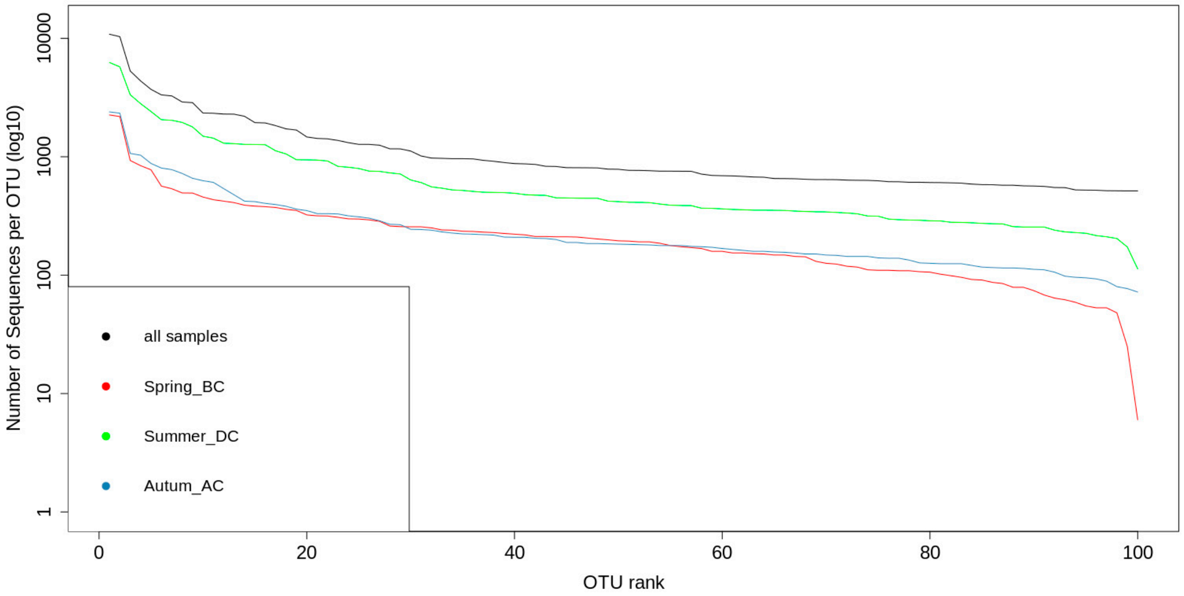

Figure S1. Rank abundance curves. Abundances of the top 100 OTUs for bacterial communities in the three seasons. Spring, summer and autumn correspond to the field conditions before, during and after cultivation of the runner bean, respectively.

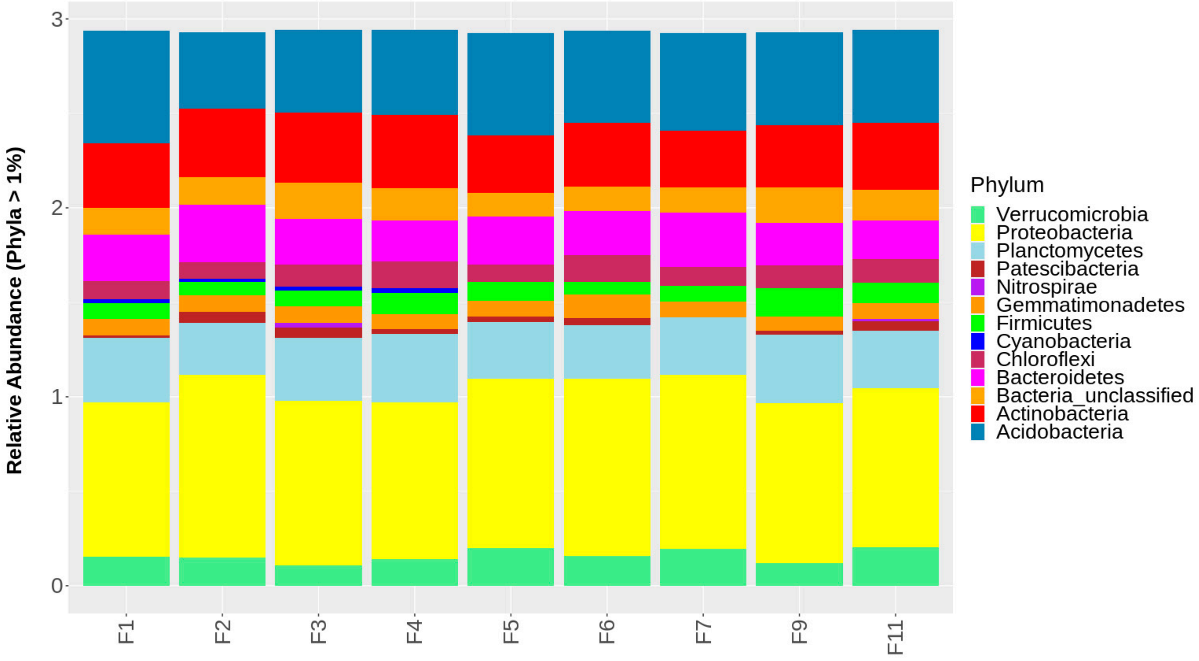

Figure S2. Abundance (%) of representative phyla of the bacterial community present in the different fields (F1-F11) across three different seasons (spring, summer, autumn). The different phyla are depicted with different colours.

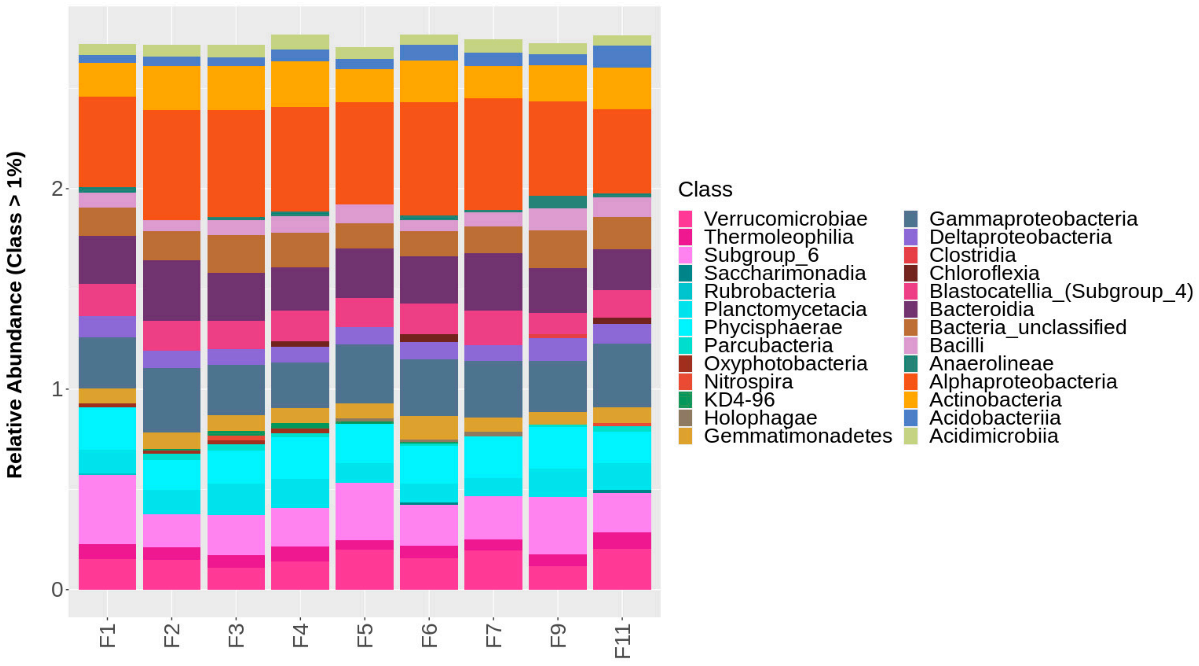

Figure S3. Abundance (%) of representative classes of the bacterial community present in the different fields (F1-F11) across three different seasons (spring, summer, autumn). The different phyla are depicted with different colours.

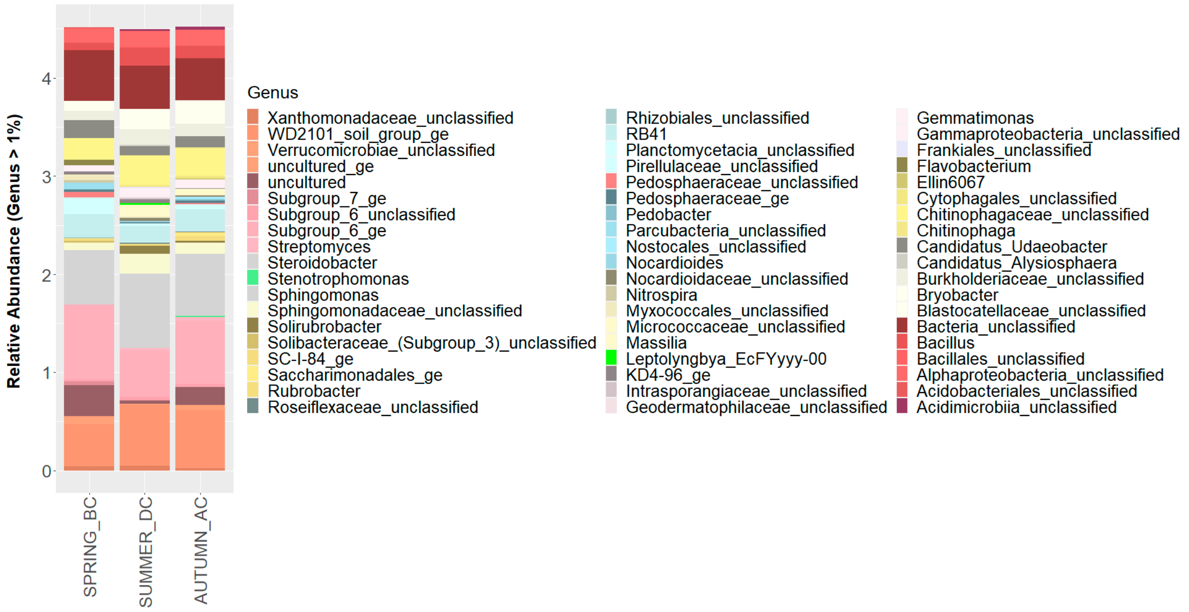

Figure S4. Classification of bacterial genera based on relative abundance at rate greater than 1% over three seasons: Spring_before cultivation (BC), Summer_during cultivation (DC), Autumn_after cultivation (AC) of the runner bean crop across the soil samples of nine field sites. The different genera are depicted in different colours.

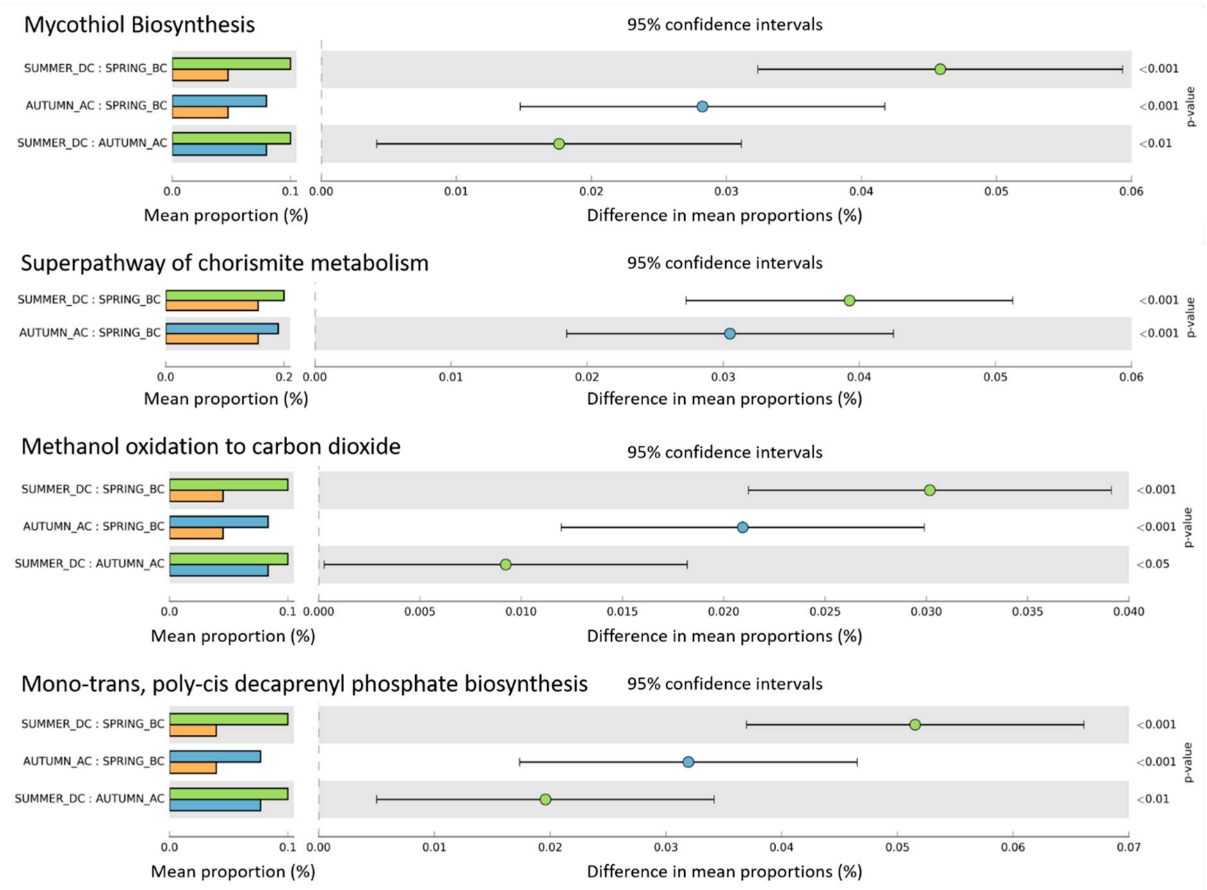

Figure S5. Post-hoc plots for the predicted pathways demonstrating greater abundance of sequences (%) in different seasons (Spring_BC, Summer_DC and Autumn_AC) across all field sites, indicating: i) the mean proportion of sequences within each season, ii) the difference in mean proportions for each pair, and iii) a *p*-value indicating whether the mean proportion is equal for a given pair. The analysis was performed in the STAMP software using ANOVA with *p*-value ≤ 0.05 and effect size >0.75.
